# Exploring the Chemoselectivity towards Cysteine Arylation by Cyclometallated Au^III^ Compounds: New Mechanistic Insights

**DOI:** 10.1002/cbic.202000262

**Published:** 2020-07-08

**Authors:** Sophie R. Thomas, Riccardo Bonsignore, Jorge Sánchez Escudero, Samuel M. Meier‐Menches, Christopher M. Brown, Michael O. Wolf, Giampaolo Barone, Louis Y. P. Luk, Angela Casini

**Affiliations:** ^1^ School of Chemistry Cardiff University Main Building Park Place CF10 3AT Cardiff UK; ^2^ Department of Analytical Chemistry Faculty of Chemistry University of Vienna Waehringer Str. 38 1090 Vienna Austria; ^3^ Department of Chemistry University of British Columbia 2036 Main Mall V6T 1Z1 Vancouver BC Canada; ^4^ Dipartimento di Scienze e Tecnologie Biologiche Chimiche e Farmaceutiche, Università degli Studi di Palermo Viale delle Scienze, Edificio 17 90128 Palermo Italy; ^5^ Department of Chemistry Technical University of Munich Lichtenbergstrasse 4 85747 Garching Germany

**Keywords:** chemoselectivity, cyclometallated gold complexes, cysteine arylation, mass spectrometry, peptides

## Abstract

To gain more insight into the factors controlling efficient cysteine arylation by cyclometallated Au^III^ complexes, the reaction between selected gold compounds and different peptides was investigated by high‐resolution liquid chromatography electrospray ionization mass spectrometry (HR‐LC‐ESI‐MS). The deduced mechanisms of C−S cross‐coupling, also supported by density functional theory (DFT) and quantum mechanics/molecular mechanics (QM/MM) calculations, evidenced the key role of secondary peptidic gold binding sites in favouring the process of reductive elimination.

## Graphical abstract

**Getting your gold right**: Cyclometallated Au^III^ complexes with bidentate C^N‐donor ligands enable chemoselective cysteine modification of peptides by C−S bond‐forming reductive elimination. By using combined high‐resolution mass spectrometry and QM/MM studies, further understanding of the mechanisms of Au^III^‐templated cross‐coupling leading to cysteine arylation was achieved; this is essential to design selective gold compounds for bio‐orthogonal reactions.

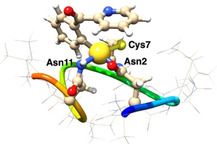

The use of metal‐based catalysts within living systems is a research area that has gained significant attention for biological sensing, imaging, caging applications and therapy.^[1]^ Among the possible reactions templated by metal compounds in biological environments, Suzuki‐Miyaura,^[2]^ Mizoroki‐Heck and Sonogashira^[3]^ cross‐coupling reactions have been described for Pd^II^ compounds and successfully applied to modify biomolecules, proteins in particular, and to study their function.^[1c]^ Recently, some examples have appeared concerning the use of gold compounds for selective bioorthogonal transformations through C−C or C−X (X=heteroatom) bond formation for different applications in chemical biology.^[1e]^ Many of the investigations exploit the propensity of both Au^III^ and Au^I^ ions to activate alkynes towards nucleophilic addition for catalysis. In this context, selective cysteine arylation using organogold reagents represents another important example of metal‐mediated bioconjugation reactions. Wong and co‐workers^[4]^ pioneered this approach forming C−S bonds on cysteine models using the cyclometallated Au^III^ C^N complex **A**, featuring a *N*,*N’*‐bis(methanesulfonyl)ethylene (msen) ligand (Figure 1) and derivatives. According to the authors, a compound's affinity for cysteine arylation was mainly determined by the presence of specific ancillary ligands coordinating Au^III^ (e. g., msen vs chlorido) while maintaining the same C^N scaffold.

**Figure 1:**
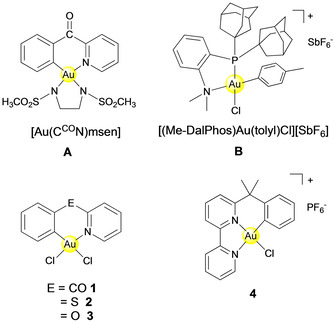
Structure of organometallic Au^III^ complexes **A** and **B** as well as of compounds **1**–**4** studied for their Cys arylation properties.

In 2018, the oxidative addition complex [(Me–DalPhos)Au(tolyl)Cl][SbF_6_] (**B**, Me–DalPhos=adamantyl_2_P(*o*‐C_6_H_4_)NMe_2_, Figure 1) was shown to arylate glutathione in aqueous environment.^[5]^ Derivatives of **B** were able to perform C−S cross‐coupling reactions with complex peptide substrates, as observed by mass spectrometry.^[5]^ However, cysteine arylation was monitored mostly at short incubation times (1 min) after addition of the Au^III^ complexes,^[5]^ which may prevent detection of other adducts and arylation products likely to occur in a complex biological environment. Further exploring the potential of C^N‐cyclometallated Au^III^ complexes for cysteine arylation in zinc finger (ZF) protein domains of the type Cys_2_His_2_,^[6]^ we demonstrated the key role of the C^N ligand in modulating the affinity of the complexes towards C−S coupling through reductive elimination.^[7]^ Among the selected Au^III^ compounds, featuring different C^N scaffolds, the [Au(C^CO^N)Cl_2_] complex (**1**, C^CO^N=2‐benzoylpyridine, Figure 1) was identified as the most prone to cysteine arylation in buffered aqueous solution (pH 7.4) at 37 °C by mass spectrometry.^[7]^ Density functional theory (DFT) and quantum mechanics/molecular mechanics (QM/MM) studies supported a mechanism which includes the following steps: i) substitution of one of the two chlorido ligands with cysteinate from the peptide, ii) apical approach of a second cysteinate residue inducing the displacement of the N atom of the C^N ligand from gold, and iii) reductive elimination leading to the C−S cross‐coupling product and to the Au^I^ side complex.^[7]^ The obtained results corroborated the idea that the specificity and efficiency in gold‐ligand binding and subsequent C−S transfer may be modulated both by the nature of the gold compound, as well as by the nucleophilicity and accessibility of the cysteines in the folded peptide, leading to stabilization of the various reaction intermediates. Notably, reductive elimination with “free” cysteine residues was never observed in aqueous solution.

Using a hyphenated mass spectrometry approach, we further explore the reactivity and chemoselectivity of cyclometallated Au^III^ complexes towards different model peptides, namely the Cys_2_His_2_ zinc finger domain (ZF), four different peptides, with and without cysteine residues available for arylation, and towards glutathione (GSH; Figure 1). The model peptides with sequences ANGELACASINI (AC), CASINI (C) and LFRANALK (L) were synthesized and characterized as described in the experimental section (Figures S1–S6 in the Supporting Information). The small library of Au^III^ compounds include cyclometallated Au^III^ complexes with bidentate C^N ligands‐**1**,^[8]^ **2** [Au(C^S^N)Cl_2_] (C^S^N=2‐(phenylthiol)pyridine)^[9]^ and **3** [Au(C^O^N)Cl_2_] (C^O^N=2‐phenoxypyridine)^[9]^ as well as with a tridentate C^N^N ligand‐**4** [Au(bipy^dmb^‐H)Cl][PF_6_] (bipy^dmb^=6‐(1,1‐dimethylbenzyl)‐2,2’‐bipyridine;^[10]^ Figure 1). The latter Au^III^ C^N^N scaffold has never been explored before for cysteine arylation. Initially, each gold complex was incubated separately with the peptides in (NH_4_)_2_CO_3_ buffer (25 mM, pH 7.4) and the samples analysed at different incubation times (30 min and 24 h at 37 °C) by high‐resolution liquid chromatography electrospray ionization mass spectrometry (HR‐LC‐ESI‐MS) following previously reported procedures[^7^, ^11^] (see also the Experimental Section for details, Figure S7–S25 and Tables S1–S6). Figures 2A and S7–S10 report representative HR‐LC‐ESI‐MS spectra obtained for complexes **1**–**4** after 30 min incubation with the ZF domain. Overall, the four compounds give classical [Apo‐ZF+Au^III^C^N‐2H]^*n*+^ adducts following chlorido ligand substitution and Zn^2+^ ejection, as previously observed for similar cyclometallated complexes;^[11]^ however, only the C^N compounds **1**–**3** produce the reductive elimination product in which the peptide is arylated at specific sites. In the case of ZF, up to two arylation sites can be detected to give [Apo‐ZF+2 C^N‐2H]^*n*+^ adducts, most likely corresponding to the two cysteine residues in the ZF domain (Table S1). Compound **3** also provides [Apo‐ZF+Au^III^C^O^N+C^O^N+3H]^6+^ adducts. Instead, complex **4** affords only bis‐adducts in which the Au^III^ centre maintains the C^N^N ligand [Apo‐ZF+2Au^III^C^N^N‐4H]^*n*+^, even after 24 h incubation. Based on the chromatographic analysis, adduct formation is never quantitative in all cases with respect to the free ZF peptide even after 24 h (as peaks corresponding to other by‐products were observed); nevertheless, complex **1** is the most reactive with the ratio of peak area of adduct to free ZF being largest (Figure S7).

**Figure 2:**
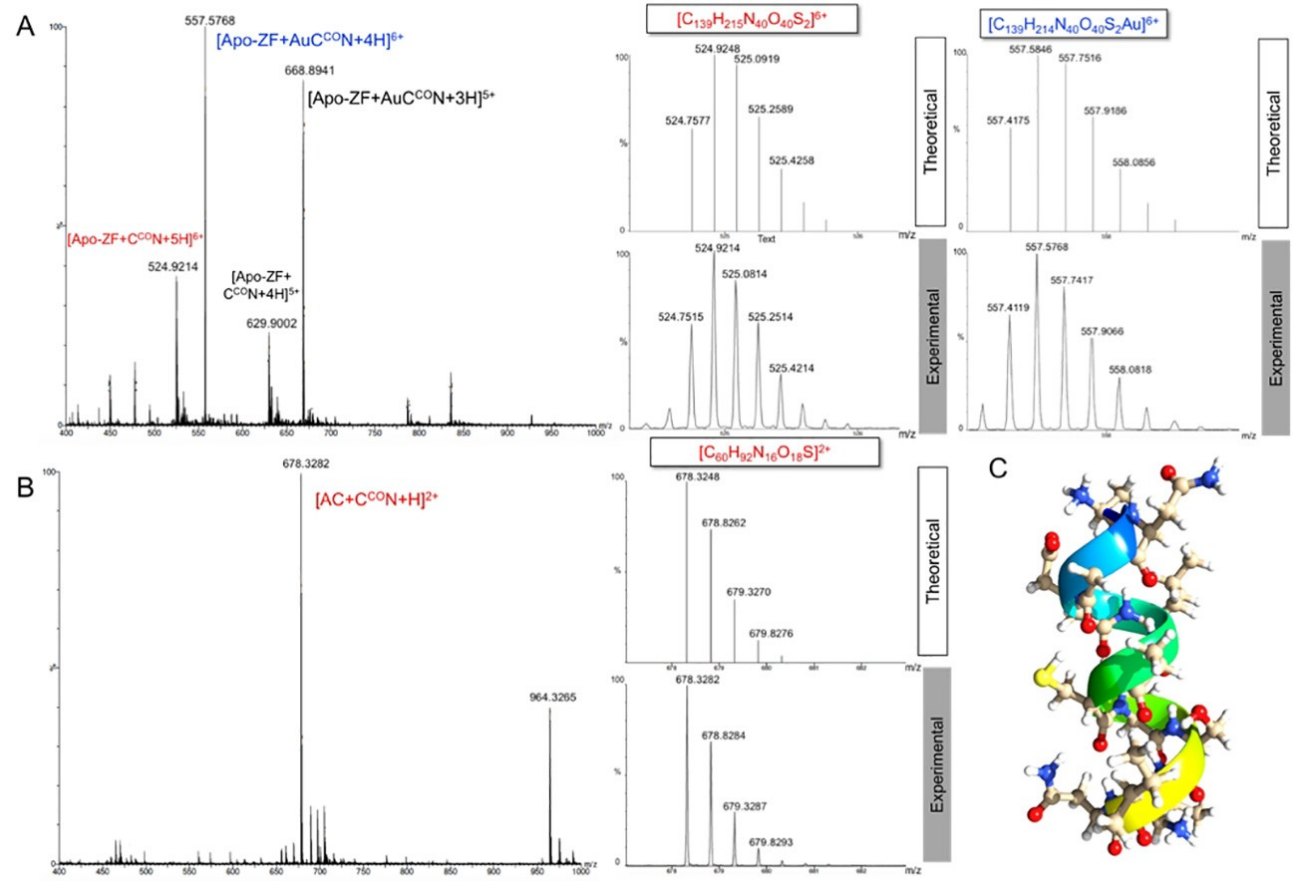
HPLC‐ESI‐MS analysis of the reaction of the Au^III^ C^CO^N complex **1** with A) ZF or B) AC peptides ((NH_4_)_2_CO_3_ buffer 25 mM, pH 7.4) after 30 min incubation at 37 °C. Representative mass spectra recorded at retention times (*t*_R_) of 3.96 and 5.32 min, respectively. Comparisons between experimental and theoretical isotopic pattern distributions for selected adducts corresponding to Cys‐arylated products. C) Model of the ANGELACASINI (AC) peptide. Drawing produced by using the UCSF Chimera package.

The ANGELACASINI (AC) peptide is very reactive with the gold complexes, particularly with compound **1** (Figure 2B and C). After 30 min incubation, the mass spectra of **1**–**3** show formation of mono‐gold [AC+Au^III^C^N‐2H]^*n*+^ and bis‐gold [AC+2Au^III^C^N‐4H]^*n*+^ adducts, reductive elimination [AC+C^N−H]^*n*+^ products, as well as their co‐existence [AC+Au^III^C^N+C^N‐3H]^*n*+^ on the same peptide molecule (Figures 2 and S11–S13, Table S2). In stark contrast, peptide arylation occurs only at one site, at variance with the case of the ZF peptide, and yet the peptide is able to offer a second Au binding site other than cysteine. After 24 h, the high reactivity of compound **1** favours complete peptide decomposition (data not shown), whereas complexes **2** and **3** still form intact [AC+Au^III^C^N+C^N−H]^2+^ adducts (Figure S12, Table S2). The cleavage of AC by **1** may occur by hydrolysis of the backbone amide bonds, as already reported for Pt^II^ and Pd^II^ complexes.^[12]^ Compound **4** does not produce arylation products, but only mono‐adducts of the type [AC+Au^III^C^N^N]^2+^ (Figure S13, Table S2). Inspection of the chromatograms shows that adduct formation is almost quantitative after 30 minutes’ incubation in all cases (data not shown).

Carrying a N‐terminal cysteine residue, the CASINI (C) peptide reacts with all compounds after 30 min forming mainly mono‐gold adducts of the type [C+Au^III^C^N−H]^+^ (**1**–**3**; Figures S14–S16, Table S3) and [C+Au^III^C^N^N−H]^+^ (**4**; Figure S17). This result is interesting as it suggests that, despite the presence of the cysteine as in peptide AC, the fact that this residue now occupies the N‐terminal position does not favour reductive elimination. In any case, compound **3** also produces [C+2Au^III^C^O^N‐3H]^+^ adducts, as detected after 30 min incubation, thus, indicating the presence of a secondary gold binding site, as in the case of the AC peptide (Figure S16, Table S3). After 24 h, the spectra show substantially the same types of adducts (Table S3).

The gold complexes were also reacted with GSH, and whereas **1**–**3** were able to fully arylate the peptide forming [GSH+C^N]^+^ adducts (Figure S18), complex **4** could only form Au^III^ adducts of the type [GSH+Au^III^C^N^N−H]^+^ even after 24 hours’ incubation (Figure S19, Table S6). In this latter case, no reduction of the Au^III^ centre could be observed despite the reducing power of GSH, in line with previous studies of **4** with other physiologically relevant reducing agents.^[13]^ In order to evaluate the chemoselectivity of the arylation product by the Au^III^ complexes, peptide LFRANALK (L) was reacted with the compounds under the same conditions.

At both 30 min and 24 h, compounds **1**–**3** give mainly mono‐gold [L+Au^III^C^N]^2+^ adducts (Figure S20–S22, Table S4), and only **3** produces adducts where the gold centre has been reduced to the type [L+Au^I^+H]^2+^. In any case, no reductive elimination product is detected in all cases. Finally, compound **4** is completely unreactive even after 24 h incubation, further demonstrating its binding preference for cysteine containing peptides.^[14]^ To confirm our observations, we also reacted the gold complexes with another cysteine‐free peptide model, namely [Leu5]‐enkephalin (YGGFL, LE). The obtained results showed that the main adduct is the mono‐gold [LE+Au^III^C^N−H]^+^ species obtained for compounds **1**–**3** (Table S5, Figures S23–S25). Similarly, complex **4** was not reactive even after 24 hours’ incubation. An overview of the types of adduct formed by complex **1** with the various peptides is included in Table 1. As LE does not offer classical nucleophilic coordination partners for the gold complexes, we aimed to identify the potential binding sites of compound **1** with the LE by using an online top‐down approach. Fragmentation experiments by collision‐induced dissociation (CID) were carried out on the [LE+Au^III^C^O^NH]^+^ adduct (*m/z* 933). This fragmentation technique generates *b* and *y* fragment ions.^[15]^ Fragment mass spectra were analysed using the online software Apm^2^s recently reported by Dyson et al.,^[16]^ which allows detection of internal fragments in addition to N‐ and C‐terminal fragments. The adduct between the pentapeptide and Au^III^C^O^N fragmented efficiently and complete sequence coverage was achieved (Figure S26, Table S7). The fragments correspond mainly to *b*‐fragments, while *y*‐fragments were not detected. Moreover, the associated *a*‐fragments were detected as well, which are formed by higher collision energies upon loss of CO from the *b*‐fragments. Overall, the results suggest two possible binding modes: the first possibility being N‐terminal coordination to the Phe1, due to fact that metallated *a/b*‐fragments were abundant. Furthermore, the two internal fragments, corresponding to a4y3 and a3y3, imply that Au^III^C^N might also be coordinated to the Gly3Phe4 fragment, most likely *via* the amide backbone.

**Table 1:** Overview of the adducts formed by compound **1** with the selected peptides and respective average masses calculated experimentally (Mr_exp_ ) and theoretically (Mr_theor_). At 24 h, decomposition of the AC peptide was recorded.

Peptide	*t*_R_	Adduct	Mr_exp_	Mr_theor_	Δ
	[min]		[Da]	[Da]	[Da]
**30 min**					
ZF	3.96	[Apo‐ZF+^CON^]	3143.51	3143.55	0.04
		[Apo‐ZF+AuC^CO^N]	3339.46	3339.51	0.05
	4.25	[Apo‐ZF+2C^CO^N]	3325.31	3325.60	0.29
AC	5.15	[AC+AuC^CO^N]	1550.62	1550.61	0.01
		[AC+AuC^CO^NCl]	1588.57	1588.60	0.03
	5.32	[AC+C^CO^N−H]	1354.65	1354.65	0.0
C	5.2	[C+AuC^CO^N]	995.35	995.34	0.01
L	4.37+4.83	[L+AuC^CO^N]	1307.62	1307.60	0.02
	5.1	[L‐NH_4_+AuC^CO^N]	1289.60	1289.57	0.03
LE	6.62	[LE+AuC^CO^N]	932.29	932.31	0.02

**24 h**					
ZF	3.64	[Apo‐ZF+C^CO^N]	3143.76	3143.55	0.21
	3.89	[Apo‐ZF+2C^CO^N]	3324.83	3324.60	0.23
C	5.2	[C+AuC^CO^N]	995.34	995.34	0.0
L	4.27‐4.64	[L+AuC^CO^N]	1307.62	1307.60	0.02
	4.74‐4.93	[L‐NH_4_+AuC^CO^N]	1289.60	1289.57	0.03
LE	6.5	[LE+AuC^CO^N]	932.29	932.31	0.02
GSH	9.65	[GSH+C^CO^N]	488.15	488.14	0.01

A competition experiment was designed to evaluate the selectivity of compound **1** for the ZF in presence of peptide LE. Thus, **1** (3 equiv.) was reacted with the two different model peptides (1 equiv. each) for 30 min and studied by HR‐LC‐ESI‐MS.^[11]^ The results show that no gold adducts were observed with LE after 30 min incubation, whereas Au^III^‐ZF adducts were identified similarly to the individual experiments, that is [Apo‐ZF+Au^III^C^CO^N‐2H]^*n*+^ (Figure S27, peak at *t*_R_=3.81 min).

An atomistic support of the experimental results was achieved by DFT calculations, which evaluated the binding energy of compound **1** upon forming monodentate adducts (by exchange of one chlorido ligand) with amino acids that possibly act as Au^III^ binding sites (see the Experimental Section for details, Figures S28–S29, Table S8). As expected based on the hard–soft acid–base (HSAB) theory, the most stable adduct is formed with the thiol of cysteine residues ([Au(C^CO^N)ClCys], Δ*G*°=−90.9 kJ/mol), followed by the N‐containing side chains of arginine ([Au(C^CO^N)ClArg]^+^, Δ*G*°=−44.5 kJ/mol) asparagine ([Au(C^CO^N)ClAsn], Δ*G*°=−42.0 kJ/mol) and glutamine (([Au(C^CO^N)ClGln], Δ*G*°=−37.5 kJ/mol). Interestingly, the compound also has weak affinity for binding to O donors in tyrosine and serine residues ([Au(C^CO^N)ClTyr/Ser] Δ*G*°=−38.3 and −35.5 kJ/mol, respectively). This data confirm the possibility for **1** to coordinate different nucleophiles.

Following the mechanistic hypothesis of cysteine arylation *via* reductive elimination in Cys_2_His_2_‐type zinc finger peptides formulated in our previous study,^[7]^ we performed DFT calculations to characterize the stability of the adduct of compound **1** with the AC peptide and to predict the reaction intermediate leading to the experimentally observed arylation product. As we observed that the cysteine residue needs to be located within an amino acid sequence to undergo reductive elimination, we hypothesize that it is crucial for the Au^III^ complex to bind in a bidentate fashion to the peptide, not necessarily involving a second cysteine residue, for the reaction to proceed by reductive elimination. Such selectivity is different from many of the popularly used reagents that typically target N‐terminal cysteine residue.^[17]^

Modelling the **1**‐AC adduct, two possible residues accessible as secondary Au^III^ binding sites were identified, namely the NH_2_ groups of the side‐chain of Asn2 and Asn11, located on both sides of the primary binding site Cys7. First, the substitution of the two chlorido ligands of **1** occurs by the binding of both Cys7 and Asn11 side‐chain residues, forming the reactant **R** in our proposed reaction pathway (Figure 3A and B). Then, the Asn2 substitution of the pyridyl ring of the C^N ligand may occur providing the intermediate **I**, which is 114 kJ/mol more stable than the reactant. Finally, two possible cross‐coupling reactions may take place, both thermodynamically favoured compared to the intermediate, and involving either i) the Asn2 nitrogen (product **P1**) or ii) the Cys7 sulfur (product **P2**; Figure 3A, B). Side products of **P1** and **P2** are the Au^I^ complexes linearly coordinated to the side chains of Asn2 and Asn11 or to Asn2 and Cys7, respectively. Interestingly, the N‐arylation product is about 20 kJ/mol more stable than the S‐arylation product (–195 vs. –175 kJ/mol, respectively). This result might be due to the increased stability of the corresponding S‐bound Au^I^ side product included in the calculations, but never observed experimentally.

**Figure 3:**
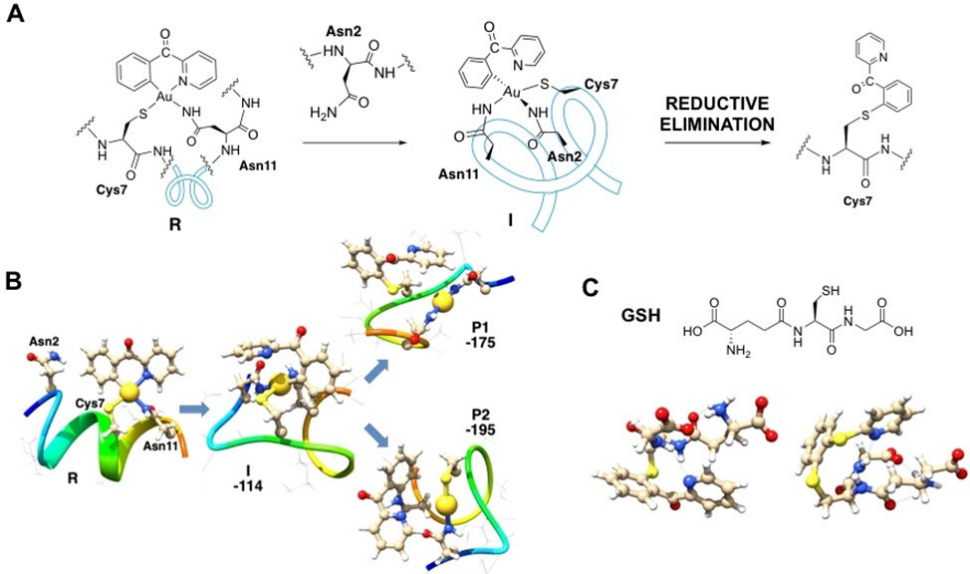
A) Proposed mechanism of cysteine arylation by the Au^III^ complex **1** reacting with the model AC peptide. B) Optimized geometries of reactant (**R**), intermediate (**I**) and products (**P1** and **P2**) obtained for the proposed mechanism by QM/MM calculations. C) Most‐stable conformers of the C−S cross‐coupling products of **1** (left) and **2** (right) with glutathione (GSH) found by DFT calculations (Figures S30 and S31). Drawings produced by using the UCSF Chimera package.

Although our MS data does not allow discrimination between the two possible arylation positions, the identification of the [AC+Au^III^C^N+C^N−H]^2+^ species for the bidentate cyclometallated complexes **2** and **3** suggests the presence of more than one binding site for Au^III^ ions relevant to the mechanism of reductive elimination other than Cys7, possibly the Asn residues themselves. In fact, a secondary gold binding site was also identified in the case of the C peptide. Moreover, the experimental observation that a cysteine in terminal position does not undergo reductive elimination (as for C) may be due to the fact that the Au^III^ centre does not find the ideal coordination environment to stabilize the intermediate **I**, leading to reductive elimination. Instead, the latter can occur even in short peptidic sequences provided that **I** can be formed, as for GSH (Figures 3C, S30 and S31). Furthermore, as observed in the case of complex **4**, if the coordination sphere of the Au^III^ centre leaves only one position suitable for peptide binding upon ligand substitution, intermediate **I** cannot be formed and the reductive elimination process does not take place. It should be noted that Gln residues may be alternative binding partners for gold, facilitating formation of **I**, as supported by DFT studies.

In conclusion, we have shown here that metal‐templated cross‐coupling reactions with peptides cannot be simply predicted on the basis of the HSAB theory and affinity of the metal for a certain nucleophile, but require a deeper understanding of the mechanisms of reaction and knowledge of the influence of the chemical and structural complexity of the target biomolecule on the overall reactivity. Such knowledge, together with the judicious choice of the ligands stabilizing the organometallic Au^III^ centre while enabling its bio‐reactivity, will certainly make gold‐promoted reactions a significant addition to the toolbox of life compatible transformations. In the future, we envisage the application of Au^III^ C^N complexes in proteomic profiling of cysteine residues and of their oxidation states,^[18]^ as well as catalysts in different cross‐coupling processes in aqueous environment.^[19]^

## Conflict of interest

The authors declare no conflict of interest.

## Supporting information

As a service to our authors and readers, this journal provides supporting information supplied by the authors. Such materials are peer reviewed and may be re‐organized for online delivery, but are not copy‐edited or typeset. Technical support issues arising from supporting information (other than missing files) should be addressed to the authors.

Supplementary
