# Supplementary material for: Exploring the Chemoselectivity towards Cysteine Arylation by Cyclometallated AuIII Compounds: New Mechanistic Insights
Source: Chembiochem. 2020 Jul 8;21(21):3071–6. doi: 10.1002/cbic.202000262 (PMC7689846; doi:10.1002/cbic.202000262)
Supplement: Supplementary file 1 — Supplementary [file CBIC-21-3071-s001.pdf]

ChemBioChem

Supporting Information

Exploring the Chemoselectivity towards Cysteine Arylation by Cyclometallated Au^{III} Compounds: New Mechanistic Insights

Sophie R. Thomas+, Riccardo Bonsignore+, Jorge Sánchez Escudero, Samuel M. Meier-Menches, Christopher M. Brown, Michael O. Wolf, Giampaolo Barone, Louis Y. P. Luk,* and Angela Casini*

Author Contributions

M.W. Investigation:Supporting; Supervision:Supporting

Supporting Information

Experimental Section

General. Solvents and reagents (reagent grade) were all commercially available and used without further purification. The zinc finger precursor peptide (ZF) was obtained from Peptide Specialty Laboratories GmbH and had the sequence [1]PYKCPECGKSFSQKSDLVKHQRTHTG[26]. Leu-enkephalin (LE) and glutathione (GSH) were purchased from Waters and Fluorochem, respectively. Ammonium carbonate, dimethylsulfoxide (DMSO), zinc acetate dihydrate, water (molecular biology grade) were purchased from Fisher. Low Loading Rink-Amide resin (Fluorochem) was used for solid phase peptide synthesis. Deionized water was obtained from an Elga PURELAB Option system (15 MΩ·cm). Fmoc-amino acids(PG)-COOH, N, N, N′, N′-tetramethyl-O-(1H-benzotriazol-1-yl) uronium hexafluorophosphate (HBTU) and hydroxybenzotriazole (HOBT), triisopropylsilane (TIS) and 3,6-dioxa-1,8-octane-dithiol (DODT) were purchased from Cambridge Reagents. N, N-Diisopropylethylamine, piperidine and trifluoroacetic acid were purchased from Fluorochem. N, N-dimethylformamide (DMF) was purchased from Cambridge Reagents and dichloromethane (DCM) from Fisher Scientific. For HPLC mobile phase, HPLC grade acetonitrile (ACN) from Fisher Scientific and trifluoroacetic acid from Fluorochem were used. Dithiothreitol (DTT) was purchased from Alfa Aesar. Compounds **1-4**, as well as the ligand of compound **2**, were synthesized adapting procedures already reported in literature,[1] and the purity of the compounds was confirmed by elemental analysis which showed purity >98%.

Peptide synthesis.

Synthesis of ANGELACASINI (AC)

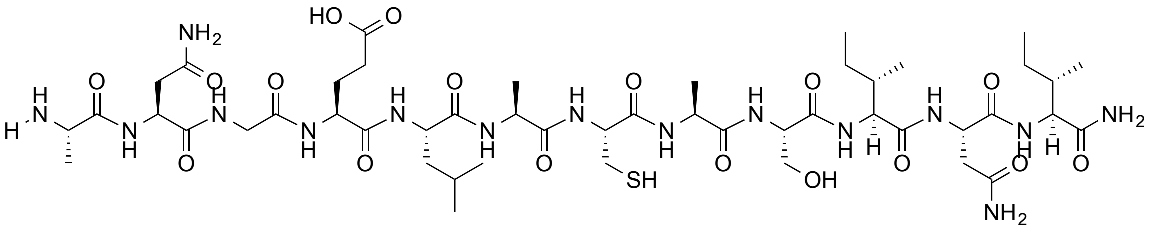

Figure S1. Structure of ANGELACASINI.

The peptide ANGELACASINI (Ala-Asn-Gly-Glu-Leu-Ala-Cys-Ala-Se-Ile-Asn-Ile, AC, Figure S1) was prepared following the solid phase peptide synthesis Fmoc protocol, using the CEM Liberty blue peptide synthesizer. The peptide was prepared on Rink amide MBHA resin (0.49 mmol·g^{-1}). Fmoc deprotection was carried out with 10% w/v piperazine in NMP:EtOH (9:1) with 0.1 M HOBt. Amino acid couplings were performed under microwave irradiation with two stages (170 W for 15 s followed by 30 W for 110 s), using 0.5 M DIC as coupling reagent and 1.0 M oxyma as additive. Two equivalents of amino acid were used per coupling cycle. Final deprotection was achieved by treating the resin with 5 mL of deprotection solution (20% v/v piperidine/DMF) twice (5 min each). The resin was then washed with DMF (15 x 5 mL) and finally diethyl ether (5 x 5 mL). The resin was dried under nitrogen and added with cleavage cocktail (10 mL 92.5:2.5:2.5:2.5 TFA:TIPS:DODT:H_2O). The mixture was stirred for 2 h. Excess TFA was removed and diethyl ether (–20 °C) added to precipitate the product. The resin was filtered off and the solution was transferred into an Eppendorf tube, centrifuged at 4000 rpm for 4 min and the solution decanted to yield the crude peptide. The crude product was dissolved in distilled water up to 1 mL and purified in a semi-preparative HPLC in water (0.1% TFA) with a gradient of increasing ACN (0.1% TFA) from 10 to 40% over 32 min (Yield = 5%, MW:1174.31 g/mol) (Figure S4).

Synthesis of CASINI (C)

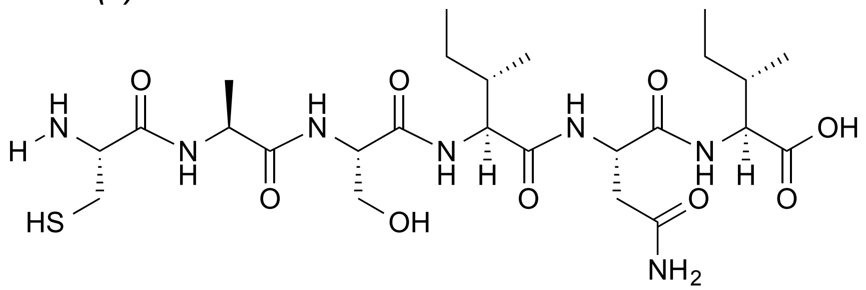

Figure S2. Structure of CASINI.

The synthesis of the peptide CASINI (Cys-Ala-Ser-Ile-Asn-Ile , C, Figure S2) was prepared following the method of solid phase peptide synthesis. In detail, 204 mg of rink amide HMBA resin (0.49 mmol·g^{-1}) was added to a peptide synthesis vessel. Following swelling (5 mL of 1:1 DMF: DCM for 30 min), the resin was treated with 5 mL deprotection solution (20% v/v piperidine/DMF) twice (5 min each). The resin was then washed with DMF (15 x 5 mL). Two equivalents Fmoc-amino acid was dissolved in a solution of HBTU (0.5 M in DMF, 0.57 mL, 0.4 eq) and HOBt (0.5 M in DMF, 0.55 mL, 3.8 eq.) that was activated by the addition of DIPEA (140 µL, 8 eq.) for 30 s. The solution was added into the resin and stirred for 40 min. The coupling procedure was repeated from the C to N terminus. Final deprotection was achieved by treating the resin with 5 mL deprotection solution twice (5 min each). The resin was washed with DMF (15 x 5 mL) and finally diethyl ether (5 x 5 mL). The resin was dried under nitrogen and subsequently added with the cleavage cocktail (10 mL 92.5:2.5:2.5:2.5 TFA:TIPS:DODT:H_2O). The mixture was left to stir magnetically for 2 h. Excess TFA was removed and diethyl ether (–20 °C) was added to precipitate the product. The resin was filtered off and the solution was transferred into an Eppendorf tube, centrifuged at 4000 rpm for 4 min and the solution decanted to yield the crude peptide. The crude product was dissolved in 1 mL distilled water and purified by HPLC in water (0.1% TFA) with a gradient of increasing ACN (0.1% TFA) from 10 to 40% over 46 min, (Yield = 7%, MW: 618.72 g/mol) (Figure S5).

Synthesis of LFRANALK (L)

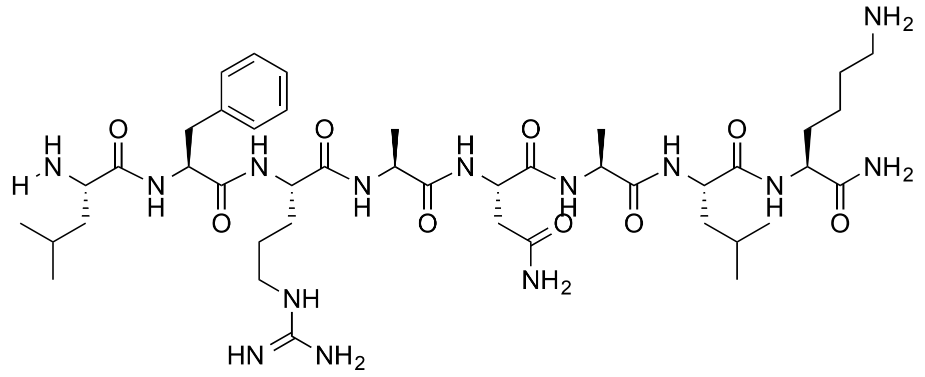

Figure S3. Structure of LFRANALK

The synthesis of the peptide LFRANALK (Leu-Phe-Arg-Ala-Asn-Ala-Leu-Lys, L, Figure S3) is following the method of solid phase peptide synthesis. 204 mg Rink amide HMBA resin (0.49 mmol g^{-1}) was weighed in a peptide synthesis vessel. Following swelling (5 mL 1:1 DMF:DCM, 30 min), the resin was filtered and treated with 5 mL 20% v/v piperidine/DMF solution twice (5 min each). The resin was then washed with DMF (15 x 5 mL). Two equivalents of Fmoc amino acid were dissolved in a solution of HBTU (0.5 M in DMF, 0.57 mL, 0.4 eq.) and HOBt (0.5 M in DMF, 0.55 mL, 3.8 eq.) and activated by DIPEA (140 µL, 8 eq.) for 30 s. The solution was then added to the resin and stirred for 40 min. The coupling procedure was repeated from C to N terminus. Final deprotection was achieved by adding 5 mL 20% v/v piperidine/DMF (2 x 5 min). The resin was washed with DMF (15x5 mL) and diethyl ether (5x5 mL). The resin was dried under nitrogen and added with the cleavage cocktail (10 mL 92.5:2.5:2.5:2.5 TFA:TIPS: DODT: H_2O). After stirring for 2 hours, TFA was removed and diethyl ether (–20 °C) was added to precipitate the product. The resin was centrifuged at 4000 rpm for 4 mins and the solution decanted to yield the crude peptide. The crude product was dissolved in 1 mL distilled water and purified by HPLC in water (0.1% TFA) with a gradient of ACN (0.1% TFA) increasing from 10-40% over 30 min, (Yield = 51.5%, MW: 931.12 g/mol) (Figure S6).

Peptide analysis.

LC-MS spectra were recorded on an Agilent Technologies 1260 LC-MS system equipped with an Agilent InfinityLab Poroshell EC-C18 column (4 µm, 4.6 x 100 mm) using 0.1% HCO_2H in water and 0.1% HCO_2H in ACN as the eluent. An Agilent Infinity 6120 Quadrupole ESI-MS module and UV detection at 210 nm were used. Data were processed using Agilent Software.

Semipreparative HPLC purification was performed using an Agilent Technologies 1260 infinity HPLC system equipped with an ACE-C18 column (5.0 µm, 10 x 250 mm) using 0.1% TFA in water and 0.1% TFA in ACN as the eluent. The products were detected 0.3 µL/min at 210 nm and data were processed using Agilent Software.

CEM Liberty Blue Automated peptide synthesizer under microwave irradiation was used to synthesize AC peptide.

UPLC-MS spectra were obtained using a Waters Synapt UPLC/MS system equipped with a Waters Acquity UPLC CSH C18 column (130 Å, 1.7 µm, 2.1 mm x 100 mm) using 0.1% HCO_2H in water and 0.1% HCO_2H in ACN as the eluent. Flow rate and column temperature were set at 0.3 µL/min and 40 °C, respectively. Mass range was set at 100-3000 m/z. The crude peptide was analyzed using a method 5-70% ACN (0.1% HCO_2H) in water (0.1% HCO_2H) over 38 min. The pure product after the purification was analyzed by LCMS using a method 5-35% ACN (0.1% HCO_2H) in water (0.1% HCO_2H) over 38 min. The pure product was analyzed by UPLC using a method water (0.1% HCO_2H) with increasing gradient of ACN (0.1% HCO_2H) from 1 to 98% over 13 min.

High-resolution mass spectra were recorded on a Waters Synapt G2-Si quadrupole time of flight mass spectrometer operated in electrospray positive ionisation mode.

HPLC-ESI-MS studies.

High Resolution HPLC-ESI-MS spectra of gold compounds/peptide adducts were recorded on Synapt G2-Si time-of-flight (TOF) mass spectrometer (Waters) by high-pressure liquid chromatography (HPLC). HPLC was performed with an Acquity UPLC system (Waters) and using an Acquity UPLC protein BEH C4 column (300 Å, 1.7 µm, 2.1 mm × 100 mm) for ZF peptide and an Acquity UPLC CSH C18 column (130 Å, 1.7 µm, 2.1 mm x 100 mm) for the remaining peptides. The instrumental parameters for high-pressure liquid chromatography mass spectrometry (HPLC-MS) were as follows: 2.85 kV capillary voltage, 120 °C source temperature, 350 °C desolvation temperature, 90 $L \cdot h^{-1}$ cone gas, 900 L·h-1 desolvation gas and 6 bar nebulizer. A linear gradient from 95% to 5% water (0.1% FA), while proportionally increasing acetonitrile (0.1% FA), in 8 min was used. The flow rate was 300 $\mu L \cdot min^{-1}$, the column was held at 40 °C and the autosampler at 20 °C. Mass spectra were acquired and processed using MassLynx V4.1 (Waters).

Solutions of L and LE were prepared by dissolving the peptides in $(NH_4)_2CO_3$ (25 mM, pH = 7.4) at 37°C. The peptides ZF, AC and C were incubated with DTT (3 eq., 3 h) in $(NH_4)_2CO_3$ (25 mM, pH = 7.4) at 37°C. The ZF peptide was reconstituted according to a previously published procedure[2] by further incubation with zinc acetate (3 eq., 30 min) at 37°C. The formation of the zinc finger was assessed by a mass shift in the resulting mass spectra. Stock solutions of the gold compounds were freshly prepared in DMSO at a concentration of 10 mM and diluted to the final concentration using $(NH_4)_2CO_3$ (25 mM, pH = 7.4). The individual experiments between the gold compounds and the peptides were performed at a molar ratio of 3:1 or 1:1 (gold complex:peptide) with the peptide at a final concentration of 10 µM. The compounds were incubated at 37 °C for 30 min and 24 h.

A competition experiment, in which gold complex 1 competed for ZF *vs* LE, was carried out at a molar ratio of 3:1:1 (Au:ZF:LE), following the same preparation method described above, using an Acquity UPLC protein BEH C4 column (300 Å, 1.7 µm, 2.1 mm × 100 mm).

Tandem mass spectrometry.

Tandem mass spectra (ESI-MS/MS or ESI-MS^2) were acquired in a HPLC-MS setup by selecting the appropriate mass signal and fragmenting the parent ions at 18–34 eV.

Computational studies.

DFT calculations, with full geometry optimization, were performed on the structures of the adducts of complex **1** [Au($C^{CO}N$)Cl_2] with different amino acids following the substitution of a chlorido ligand (Figure S28-S29) and of the cross-coupling products obtained by the reaction of compounds **1** and **2** with GSH (Figures S30 and S31), by following computational approaches and models recently reported.[2b, 3] In detail, the M06-L DFT functional,[4] the Lanl2tz(f)[5] basis set for Au and the 6-311G(d,p)[6] basis set for the other atoms, were used. Water solvent effects were implicitly evaluated by the polarizable continuum model (PCM).[7] Transition state structures were located by the synchronous transit guided quasi-Newton method.[8] Vibration frequency analysis, within the harmonic approximation, was performed on each optimized geometry, to check whether it matched with an energy minimum or to a first-order saddle point (for transition state structures) in the potential energy surface, and to evaluate their standard Gibbs free energy values, at 298.15 K.

The standard formation Gibbs free energy values of the adducts of **1** [Au($C^{CO}N$)Cl_2], reported in Table S8, were obtained by hypothesizing the occurrence of the following reaction:

$$[Au(C^{CO}N)Cl_2] + AA^n \rightarrow [Au(C^{CO}N)(AA)Cl]^{(1-n)} + Cl^-$$

where n (0 or -1), is the charge of amino acid ligand, and calculated by the Eq. 1 below, where G° is the standard Gibbs free energy calculated in water solution:

$$\Delta G° = G°[Au(C^{CO}N)(AA)Cl] + G°[Cl^-] - G°[Au(C^{CO}N)Cl_2] – G°[AA] \qquad \text{Eq. 1}$$

Quantum mechanics/molecular mechanics (QM/MM) calculations have been performed to describe the binding of the Au(III) complex, and of the species along the cross coupling reaction pathway, with the ANGELACASINI peptide. The M06-L DFT functional was used in the QM layer (atoms in balls and sticks in Figure 3), composed by the Asn2, Cys7 and Asn11 residues of the peptide and by the gold compound. The UFF force field[9] was used in the MM layer (atoms in wires). Full geometry optimization was followed by a frequency analysis, to confirm that the obtained structure corresponded to an energy minimum in the potential energy surface. All calculations were performed by the Gaussian 09 program package.[10] All pictures of the molecular models were produced by the UCSF Chimera software.[11]

Tables

ZF Cys_2His_2

Peptide	RT (mins)	Species	M_{exp}	M_{theor}	Δppm
ZF Cys_2His_2	3.28	[Apo-ZF+6H]$^{6+}$	494.7449	494.7494	9.10

Table S1 – Experimental (M_{exp}) and theoretical (M_{theor}) masses of the detected species during the individual HPLC-ESI-MS experiment of Cys_2His_2 model peptide.

30 min incubation

Compound	RT (mins)	Species	M_{exp}	M_{theor}	Δppm
1	3.96	[Apo-ZF+AuC^{CO}N+4H]$^{6+}$	557.5768	557.5846	13.99
		[Apo-ZF+C^{CO}N+5H]$^{6+}$	524.9214	524.9248	6.48
	4.25	[Apo-ZF+2C^{CO}N+4H]$^{6+}$	555.2608	555.2673	11.71
2	4.03	[Apo-ZF+C^{S}N+5H]$^{6+}$	525.5815	525.5876	11.61
		[Apo-ZF+AuC^{S}N+4H]$^{6+}$	558.2365	558.2474	19.52
	4.39	[Apo-ZF+2C^{S}N+4H]$^{6+}$	556.5881	556.5930	8.80
3	3.74	[Apo-ZF+AuC^{O}N+4H]$^{6+}$	555.5796	555.5846	9.00
		[Apo-ZF+C^{O}N+5H]$^{6+}$	523.0834	523.0919	16.25
	3.91	[Apo-ZF+AuC^{O}N+C^{O}N+3H]$^{6+}$	583.7609	583.7601	1.37
	4.02	[Apo-ZF+2C^{O}N+4H]$^{6+}$	551.0939	551.1003	11.61
		[Apo-ZF+AuC^{O}N+C^{O}N+3H]$^{6+}$	583.9191	583.9271	13.70
		[Apo-ZF+AuC^{O}N+Au^{I}+3H]$^{6+}$	588.2609	588.2444	28.05
4	3.86	[Apo-ZF+2AuCNN+2H]$^{6+}$	650.9475	650.9465	1.54

24 h incubation

Compound	RT (mins)	Species	M_{exp}	M_{theor}	Δppm
1	3.64	[Apo-ZF+C^{CO}N+5H]$^{6+}$	524.9178	524.9248	13.33
	3.89	[Apo-ZF+2C^{CO}N+4H]$^{6+}$	555.0999	555.1003	0.72
2	4.03	[Apo-ZF+C^{S}N+5H]$^{6+}$	525.7515	525.7546	5.90
		[Apo-ZF+AuC^{S}N+4H]$^{6+}$	558.4117	558.4144	4.83
	4.39	[Apo-ZF+2C^{S}N+4H]$^{6+}$	556.5881	556.5930	8.80
3	3.66	[Apo-ZF+C^{O}N+5H]$^{6+}$	522.9238	522.9248	1.91
		[Apo-ZF+AuC^{O}N+4H]$^{6+}$	555.5796	555.5846	9.00
	4.00	[Apo-ZF+2C^{O}N+4H]$^{6+}$	551.2681	551.2673	1.45
4	3.86	[Apo-ZF+2AuCNN+2H]$^{6+}$	650.9475	650.9465	1.54

AC

Peptide	RT (mins)	Species	M_{exp}	M_{theor}	Δppm
AC	4.86	$[AC+H]^{+}$	1174.6035	1174.5890	12.34

Table S2 – Experimental (M_{exp}) and theoretical (M_{theor}) masses of the detected species during the individual HPLC-ESI-MS experiment of AC model peptide.

30 min incubation

Compound	RT (mins)	Species	M_{exp}	M_{theor}	Δppm
1	5.15	$[AC+AuC^{CO}N]^{2+}$	776.3030	776.3042	1.55
		$[AC+AuC^{CO}NCl+3H]^{2+}$	795.2873	795.3004	16.47
	5.32	$[AC+C^{CO}N+1H]^{2+}$	678.3282	678.3248	5.01
2	5.25	$[AC+AuC^{S}N]^{2+}$	778.2983	778.2927	7.19
	5.44	$[AC+C^{S}N+1H]^{2+}$	680.3186	680.3134	7.64
		$[AC+2AuC^{S}N-2H]^{2+}$	968.7899	968.7870	2.99
3	6.28	$[AC+2AuC^{O}N-2H]^{2+}$	952.8157	952.8099	6.09
	6.60	$[AC+AuC^{O}N+C^{O}N-1H]^{2+}$	854.8404	854.8306	11.46
4	4.78	$[AC+AuCNN]^{2+}$	821.8527	821.8434	11.32

24 h incubation

Compound	RT (mins)	Species	M_{exp}	M_{theor}	Δppm
2	6.53	$[AC+2AuC^{S}N-2H]^{2+}$	968.7899	968.7870	2.99
		$[AC+AuC^{S}N+C^{S}N-1H]^{2+}$	870.8113	870.8077	4.13
3	6.43	$[AC+AuC^{O}N+C^{O}N-1H]^{2+}$	854.8404	854.8306	11.46
4	4.80	$[AC+AuCNN]^{2+}$	821.8527	821.8434	11.32

C

Peptide	RT (mins)	Species	M_{exp}	M_{theor}	Δppm
C	4.17	$[C+H]^+$	619.3216	619.3237	3.39

Table S3 – Experimental (M_{exp}) and theoretical (M_{theor}) masses of the detected species during the individual HPLC-ESI-MS experiment of C model peptide.

30 min incubation

Compound	RT (mins)	Species	M_{exp}	M_{theor}	Δppm
1	5.20	$[C+AuC^{CO}N-1H]^+$	996.3513	996.3353	16.06
2	5.29	$[C+AuC^{S}N-1H]^+$	1000.3221	1000.3124	9.70
3	5.19	$[C+AuC^{O}N-1H]^+$	984.3491	984.3353	14.02
	6.89	$[C+2AuC^{O}N-3H]^+$	1349.3585	1349.3467	8.72
4	4.78	$[C+AuCNN-1H]^+$	1087.4188	1087.4138	4.60

24 h incubation

Compound	RT (mins)	Species	M_{exp}	M_{theor}	Δppm
1	4.98	$[C+AuC^{CO}N-1H]^+$	996.3513	996.3353	16.06
2	5.09	$[C+AuC^{S}N-1H]^+$	1000.3221	1000.3124	9.70
3	5.02	$[C+AuC^{O}N-1H]^+$	984.3491	984.3353	14.02
4	4.95	$[C+AuCNN-1H]^+$	1087.4332	1087.4138	17.84

L

Peptide	RT (mins)	Species	M_{exp}	M_{theor}	Δppm
L	3.91	$[L+H]^{+}$	931.5858	931.5842	1.72

Table S4 – Experimental (M_{exp}) and theoretical (M_{theor}) masses of the detected species during the individual HPLC-ESI-MS experiment of L model peptide.

30 min incubation

Compound	RT (mins)	Species	M_{exp}	M_{theor}	Δppm
1	4.37+4.83	$[L+AuC^{CO}N]^{2+}$	654.8054	654.8018	5.50
	4.51	$[L-NH_4+OH+AuC^{CO}N-H]^{2+}$	654.3141	654.2859	43.10
	5.1	$[L-NH_4+AuC^{CO}N]^{2+}$	645.8030	645.7845	28.65
2	4.51+4.98	$[L+AuC^{S}N]^{2+}$	656.7945	656.7903	6.40
3	4.39	$[L+AuC^{O}N]^{2+}$	648.8005	648.8018	2.00
	4.90	$[L+AuC^{O}N]^{2+}$	648.8116	648.8018	15.10
	5.10	$[L+Au^{I}+1H]^{2+}$	564.2738	564.2753	2.66

24 h incubation

Compound	RT (mins)	Species	M_{exp}	M_{theor}	Δppm
1	4.27-4.64	$[L+AuC^{CO}N]^{2+}$	654.8054	654.8018	5.50
	4.74-4.93	$[L-NH_4+AuC^{CO}N]^{2+}$	645.8030	645.7845	28.65
2	4.25+4.76	$[L+AuC^{S}N]^{2+}$	656.7945	656.7903	6.40
3	4.69	$[L+AuC^{O}N]^{2+}$	648.8228	648.8018	32.37
	4.91	$[L+Au^{I}+1H]^{2+}$	564.2738	564.2753	2.66

LE

Peptide	RT (mins)	Species	M_{exp}	M_{theor}	Δppm
LE	4.95	$[LE+H]^+$	556.2792	556.3009	39.01

Table S5 – Experimental (M_{exp}) and theoretical (M_{theor}) masses of the detected species during the individual HPLC-ESI-MS experiment of LE model peptide.

30 min incubation

Compound	RT (mins)	Species	M_{exp}	M_{theor}	Δppm
1	5.44-5.63	$[LE\text{-}NH_4\text{+}OH\text{+}AuC^{CO}N\text{-}2H]^+$	932.3051	932.2808	26.06
	6.62	$[LE\text{+}AuC^{CO}N\text{-}1H]^+$	933.2914	933.3124	22.50
	6.97	$[LE\text{-}NH_4\text{+}AuC^{CO}N\text{-}1H]^+$	915.2888	915.2781	11.69
2	5.44	$[LE\text{+}AuC^{S}N\text{-}1H]^+$	937.2682	937.2896	22.83
3	5.37+6.84	$[LE\text{+}AuC^{O}N\text{-}1H]^+$	921.2922	921.3124	21.92

24 h incubation

Compound	RT (mins)	Species	M_{exp}	M_{theor}	Δppm
1	5.22-5.39	$[LE\text{-}NH_4\text{+}OH\ \text{+}AuC^{CO}N\text{-}2H]^+$	932.3184	932.2808	40.33
	6.50	$[LE\text{+}AuC^{CO}N\text{-}1H]^+$	933.2914	933.3124	22.50
	6.92	$[LE\text{-}NH_4\text{+}AuC^{CO}N\text{-}1H]^+$	915.2888	915.2781	11.69
2	5.27+6.87	$[LE\text{+}AuC^{S}N\text{-}1H]^+$	937.2682	937.2896	22.83
3	5.22+6.75	$[LE\text{+}AuC^{O}N\text{-}1H]^+$	921.2922	921.3124	21.92

GSH

Peptide	RT (mins)	Species	M_{exp}	M_{theor}	Δppm
GSH	1.02	$[GSH+H]^+$	308.0917	308.0916	0.32

Table S6 – Experimental (M_{exp}) and theoretical (M_{theor}) masses of the detected species during the individual HPLC-ESI-MS experiment of GSH.

<u>24 h incubation</u>

Compound	RT (mins)	Species	M_{exp}	M_{theor}	Δppm
1	9.65	$[GSH+C^{CO}N]^+$	489.1411	489.1444	6.75
2	11.58	$[GSH+C^{S}N]^+$	493.1254	493.1216	7.71
3	10.85	$[GSH+AuC^{O}N-1H]^+$	673.1104	673.1031	10.84
	11.02	$[GSH+C^{O}N]^+$	477.1422	477.1444	4.61
		$[GSH+AuC^{O}N-1H]^+$	673.1104	673.1031	10.84
4	9.65	$[GSH+AuCNN-1H]^+$	776.1815	776.1817	0.26

Table S7. List of identified metalated peptide fragments from online MS/MS experiments of the $[LE+Au^{III}C\text{^}N\text{-}2H^+]^+$ adduct (*m/z* 933). The similarity score takes into account the convergence of the experimental and the theoretical isotopic distribution and mass accuracy.

Peptide	Modification	Type	M_{exp}	M_{theor}	Similarity	Charge	Intensity [%]	Δppm
YGGFL	Au^{III}C^N	parent mass	933.2886	933.2881	95	1	30	4.0
YGGFL	Au^{III}C^N	b4	802.1958	802.1935	94	1	26	3.0
YGGFL	Au^{III}C^N	a4	774.2025	774.1985	95	1	1	5.2
YGGFL	Au^{III}C^N	b3	655.1257	655.1250	96	1	11	0.9
YGGFL	Au^{III}C^N	a3	627.1311	627.1301	95	1	4	1.5
YGGFL	Au^{III}C^N	b2	598.102	598.1036	94	1	20	1.0
YGGFL	Au^{III}C^N	a2	570.1099	570.1087	87	1	6	2.2
YGGFL	Au^{III}C^N	a4y3	554.1143	554.1137	80	1	4	0.9
YGGFL	Au^{III}C^N	b1	541.0807	541.0821	66	1	1	2.6
YGGFL	Au^{III}C^N	a1	513.0863	513.0872	96	1	15	1.8
YGGFL	Au^{III}C^N	a3y3	407.0453	407.0453	84	1	1	0.1
YGGFL	Au^{I}	b4	619.1262	619.1256	n.a.	1	100	0.6

Table S8. Standard formation Gibbs free energy in water solution, ΔG° in kJ/mol, of the adducts of **1** [Au(C^{CO}N)Cl$_2$] with the indicated amino acids, obtained by DFT calculations and using Eq. 1.

**Complex**	**ΔG°** (kJ/mol)
Au(C^{CO}N)ClCys	-90.9
Au(C^{CO}N)ClAsp	-35.1
[Au(C^{CO}N)ClHis]$^+$	4.5
Au(C^{CO}N)ClSer	-35.5
Au(C^{CO}N)ClTyr	-38.3
[Au(C^{CO}N)ClArg]$^+$	-44.5
Au(C^{CO}N)ClAsn	-42.0
Au(C^{CO}N)ClGln	-37.5
[Au(C^{CO}N)ClLys]$^+$	-3.9

Figures

Figure S4. LC-MS trace of ANGELACASINI peptide.

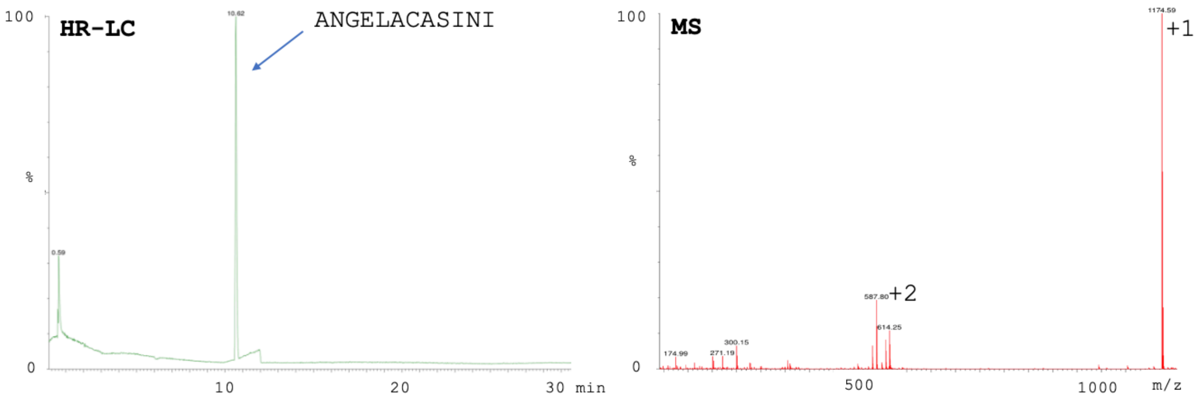

Figure S5. LC-MS trace of CASINI peptide.

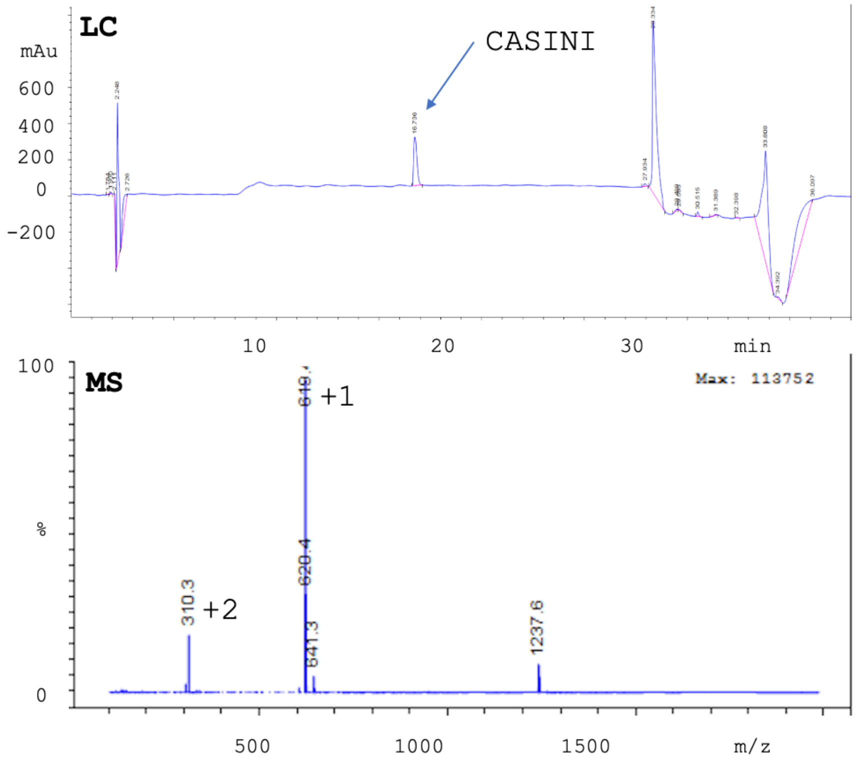

Figure S6. LC-MS trace of LFRANALK peptide.

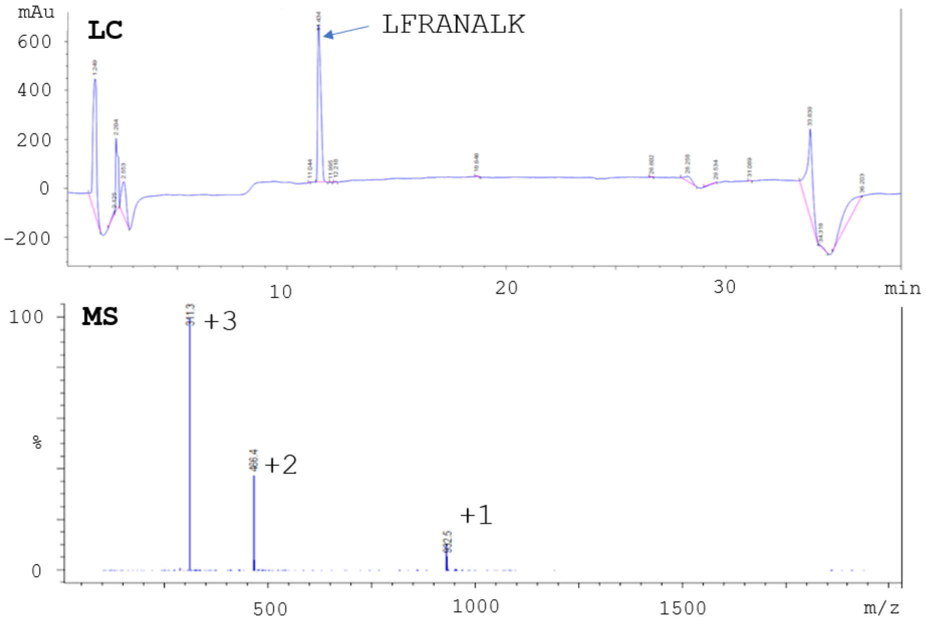

Figure S7 – Representative chromatograms of the individual HPLC-ESI-MS experiments of Cys_2His_2 with each Au(III) complex in a 1:3 ratio for either 30 min (left) or 24 h (right) incubation at 37 °C. The ‘star’ labelled peaks correspond to the free peptide, while the ‘square’ labelled peaks correspond to the analysed Au complex/peptide adducts.

Compound 1

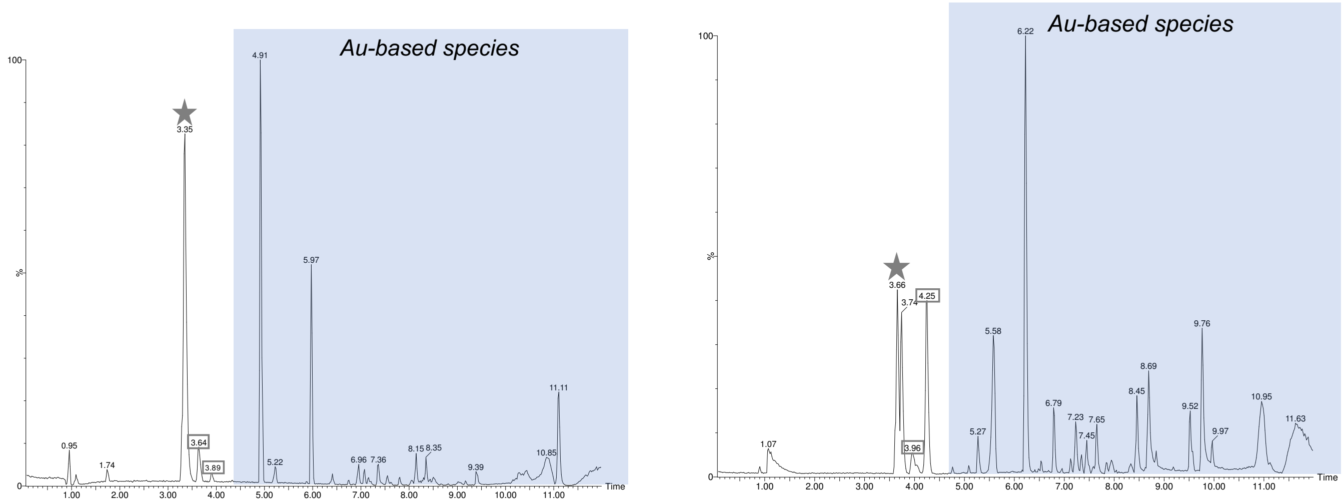

Compound 2

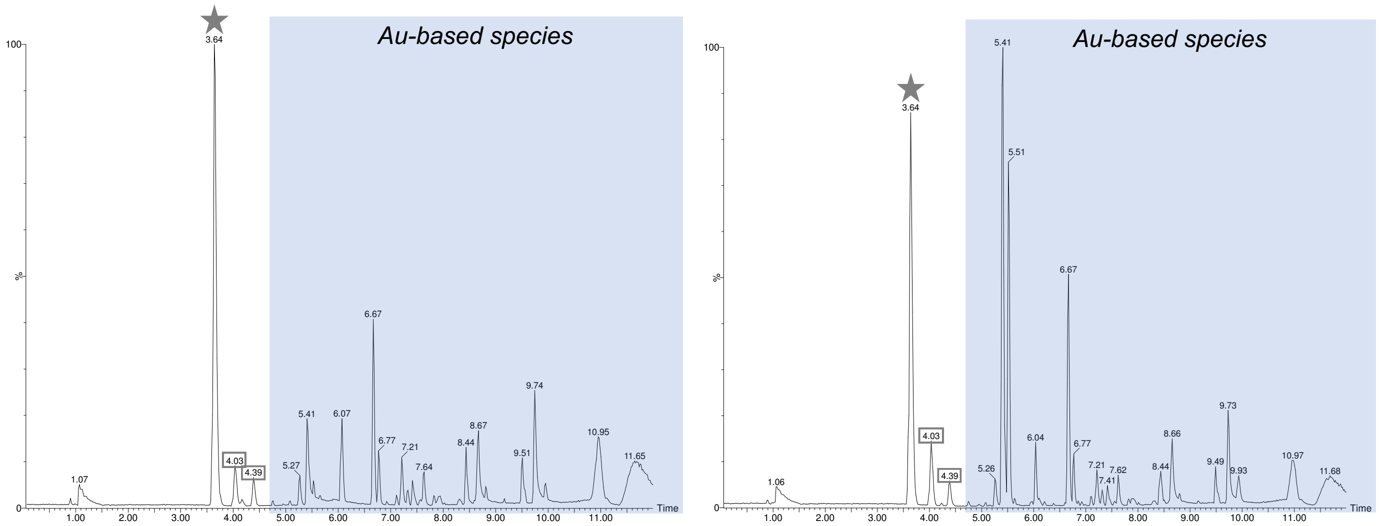

Compound 3

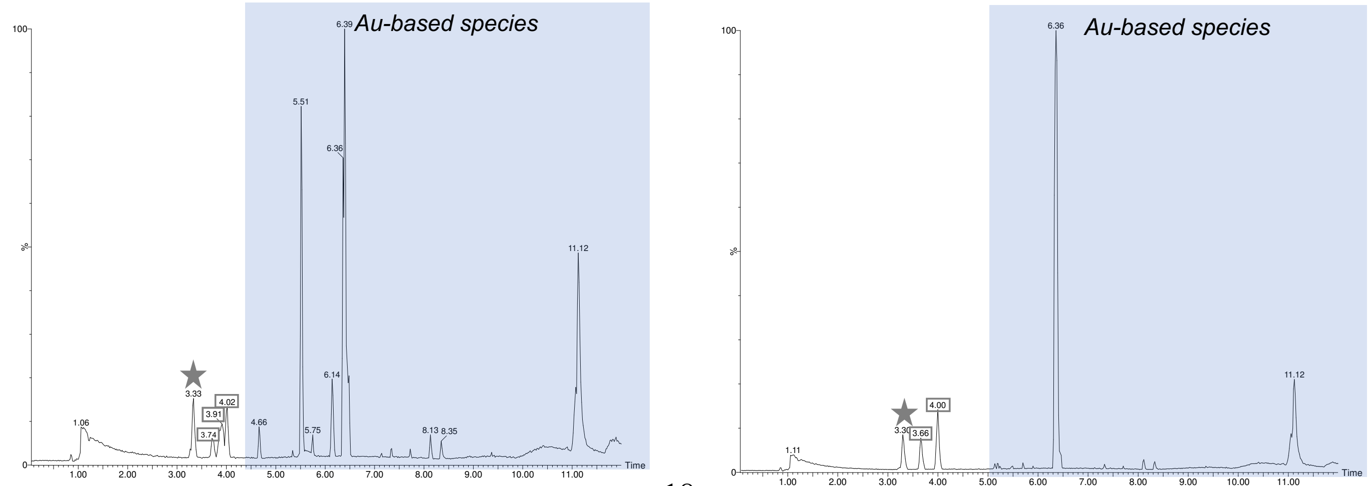

Compound 4

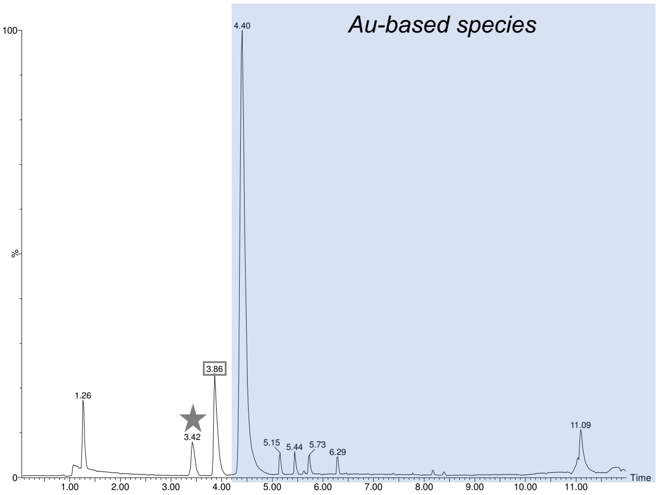
Au-based species
100
%
0
1.26
3.42
3.86
4.40
5.15
5.44
5.73
6.29
11.09
1.00
2.00
3.00
4.00
5.00
6.00
7.00
8.00
9.00
10.00
11.00
Time

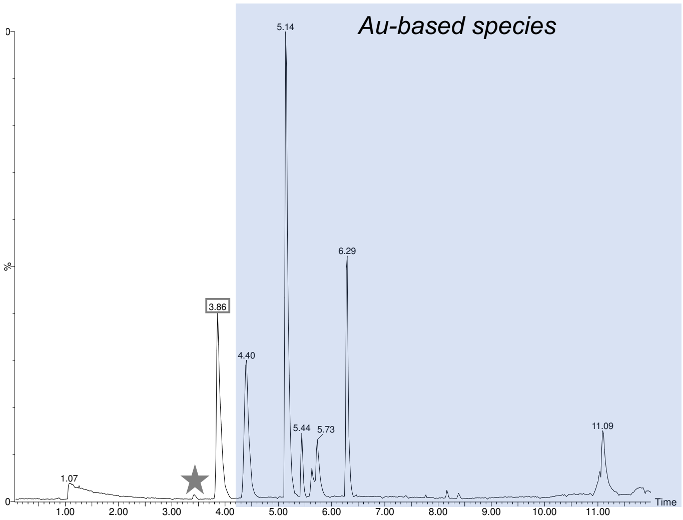
Au-based species
%
0
1.07
3.86
4.40
5.14
5.44
5.73
6.29
11.09
1.00
2.00
3.00
4.00
5.00
6.00
7.00
8.00
9.00
10.00
11.00
Time

Figure S8 - HPLC-ESI-MS analysis of the reaction of compound **2** with the ZF Cys_2His_2 model peptide (3 : 1 ratio) after 30 min incubation at 37 °C recorded at different retention times (RTs). Comparison between the experimental isotopic patterns of representative adducts with the theoretical values.

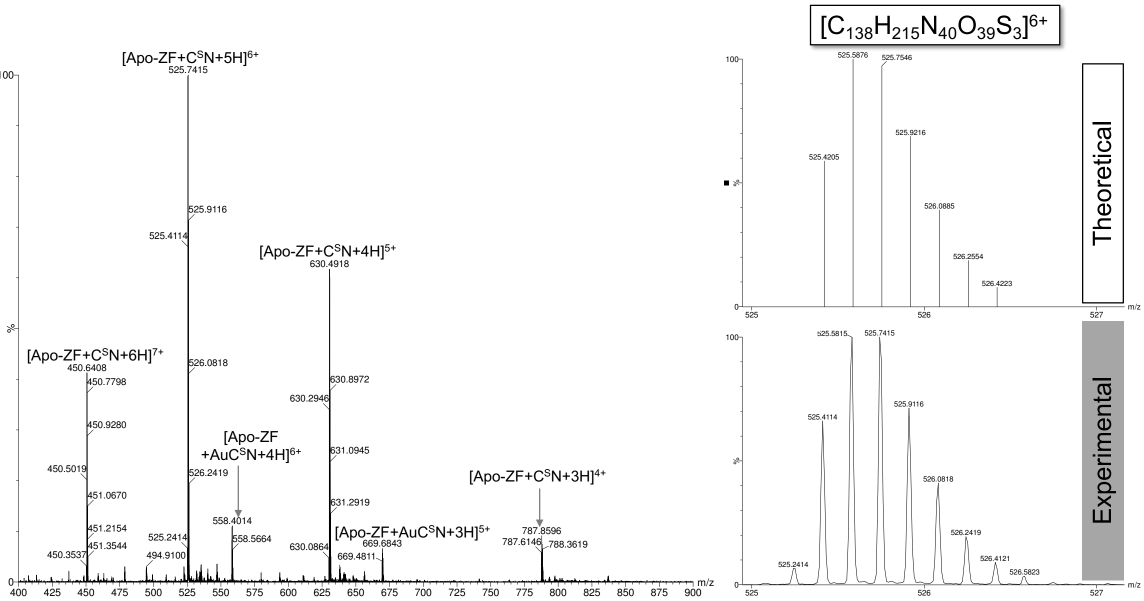

RT 4.03 min

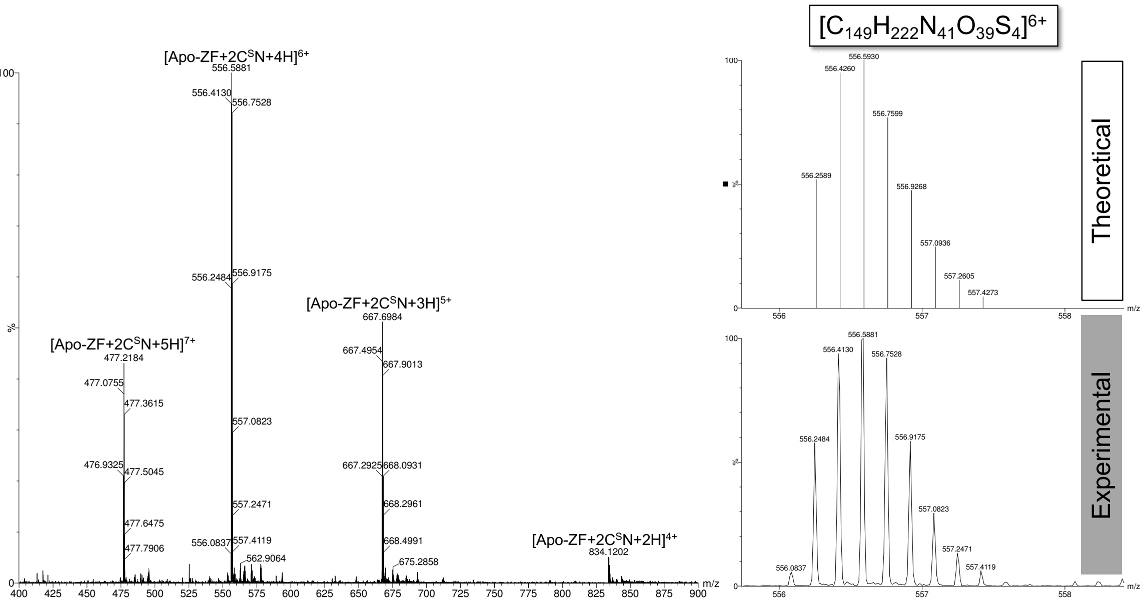

RT 4.39 min

Figure S9 - HPLC-ESI-MS analysis of the reaction of compound **3** with the ZF Cys_2His_2 model peptide (3 : 1 ratio) after 30 min incubation at 37 °C recorded at different retention times (RTs). Comparison between the experimental isotopic patterns of representative adducts with the theoretical values.

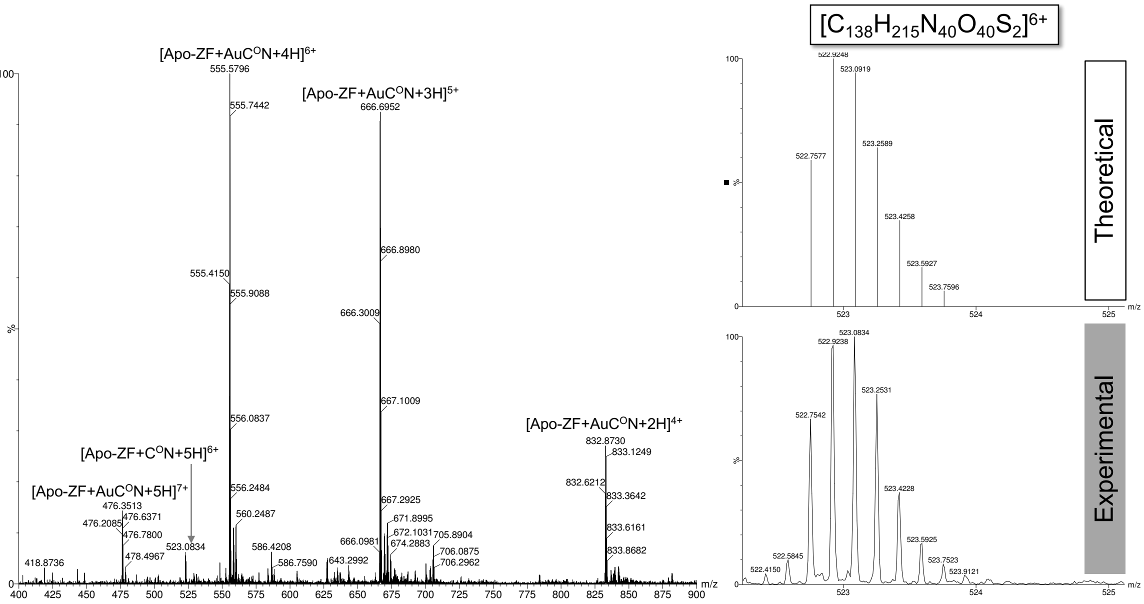

RT 3.74 min

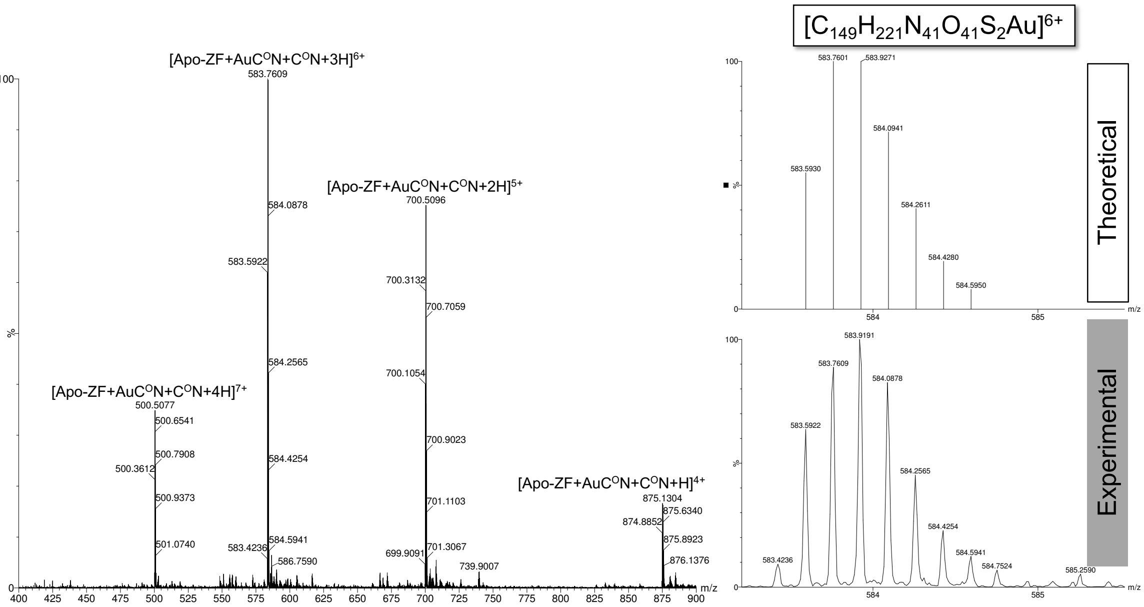

RT 3.91 min

Figure S10 - HPLC-ESI-MS analysis of the reaction of compound **4** with the ZF Cys_2His_2 model peptide (3 : 1 ratio) after 30 min incubation at 37 °C recorded at retention time 3.86 min. Comparison between the experimental isotopic patterns of representative adducts with the theoretical values.

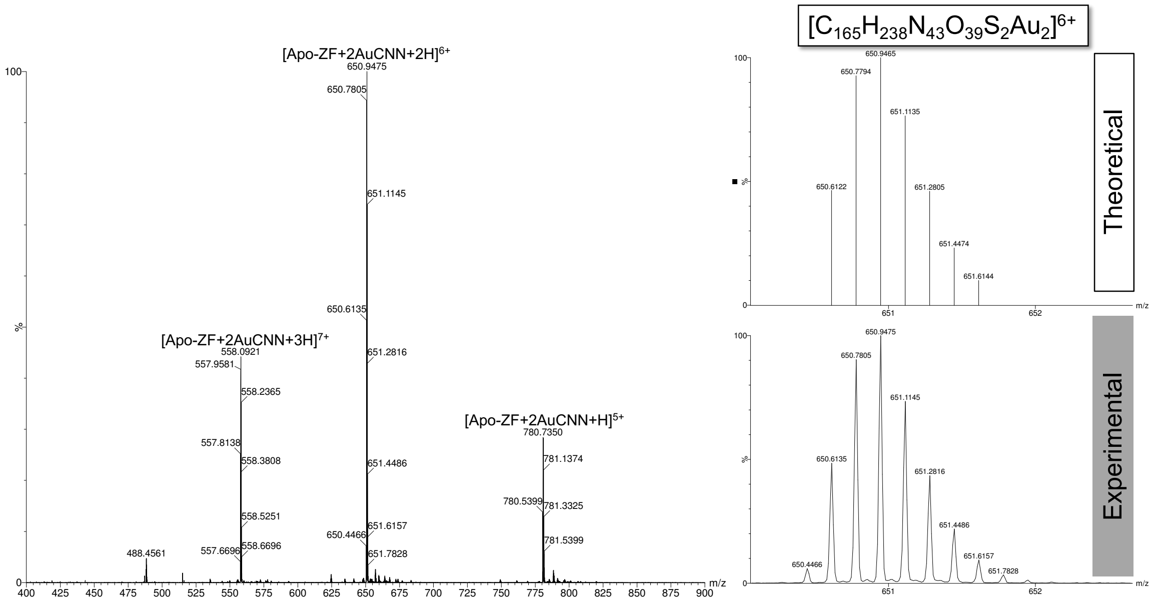

Figure S11 - HPLC-ESI-MS analysis of the reaction of compound **2** with AC model peptide (1 : 1 ratio) after 30 min incubation at 37 °C recorded at different retention times (RTs). Comparison between the experimental isotopic patterns of representative adducts with the theoretical values.

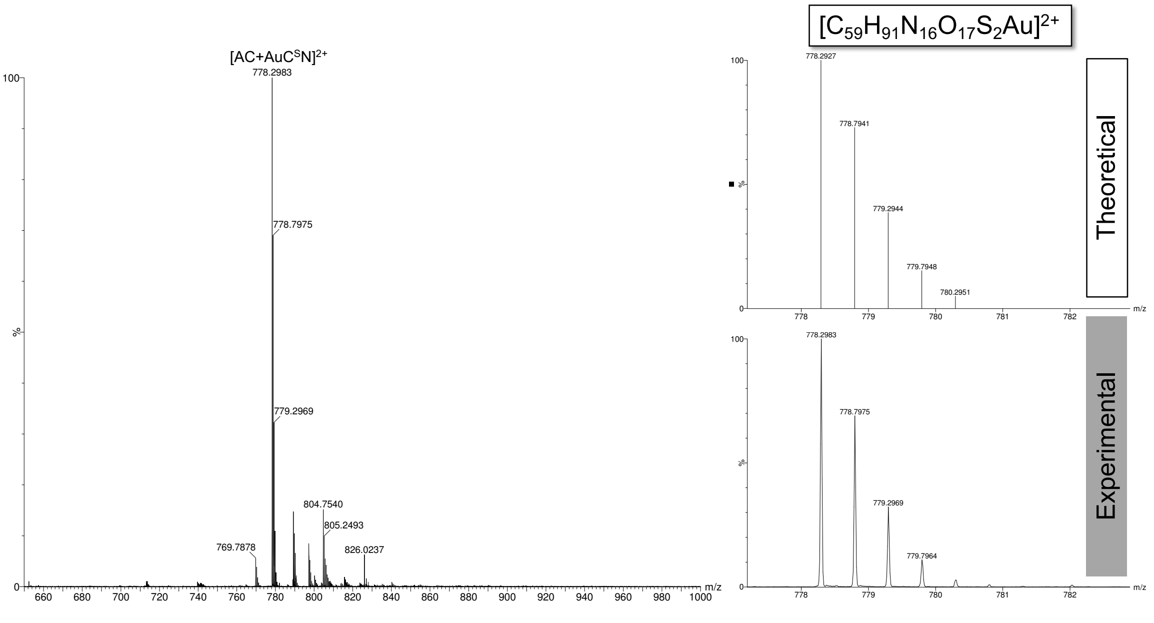

RT 5.25 min

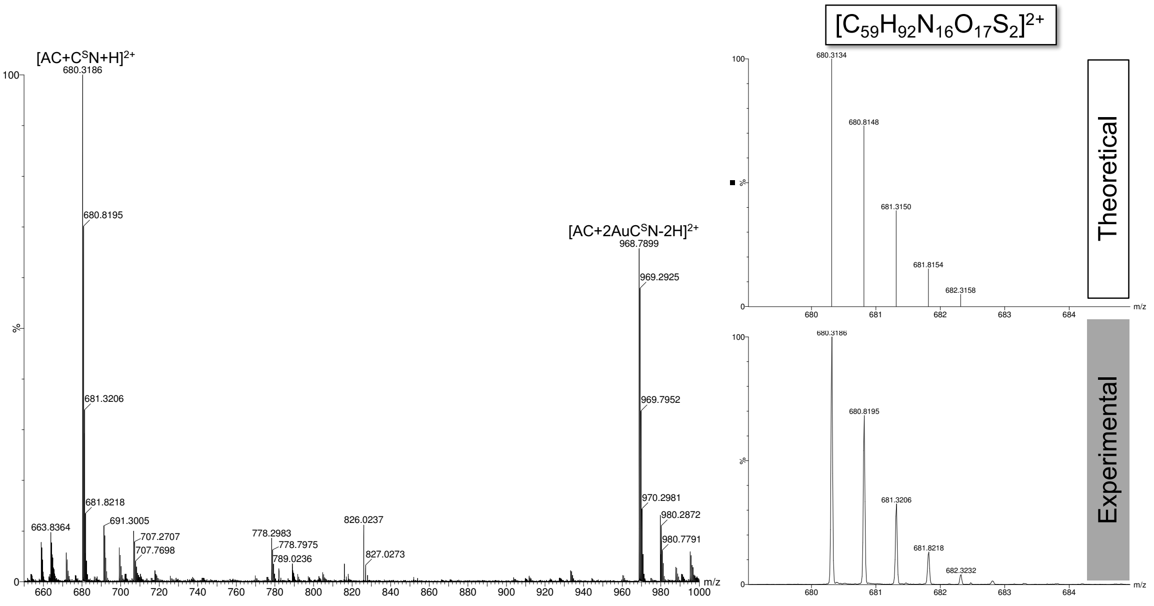

RT 5.44 min

Figure S12 - HPLC-ESI-MS analysis of the reaction of compound **3** with AC model peptide (1 : 1 ratio) after 30 min incubation at 37 °C recorded at retention time 6.60 min. Comparison between the experimental isotopic patterns of representative adducts with the theoretical values.

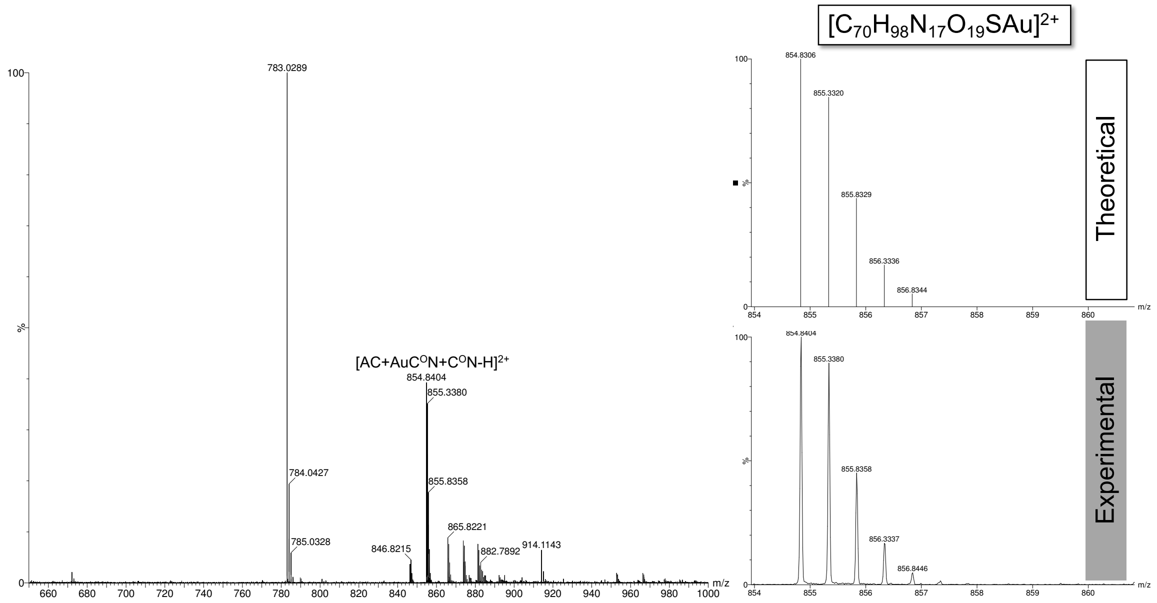

Figure S13 - HPLC-ESI-MS analysis of the reaction of compound **4** with AC model peptide (1 : 1 ratio) after 30 min incubation at 37 °C recorded at retention time 4.78 min. Comparison between the experimental isotopic patterns of representative adducts with the theoretical values.

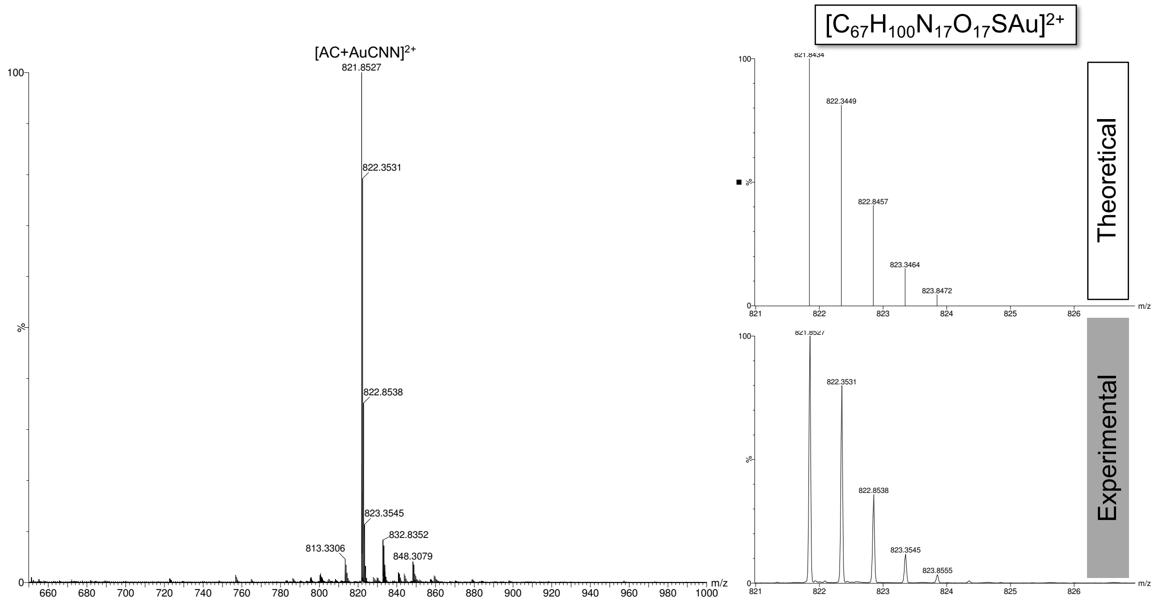

Figure S14 - HPLC-ESI-MS analysis of the reaction of compound **1** with C model peptide (1 : 1 ratio) after 30 min incubation at 37 °C recorded at retention time 5.20 min. Comparison between the experimental isotopic patterns of representative adducts with the theoretical values.

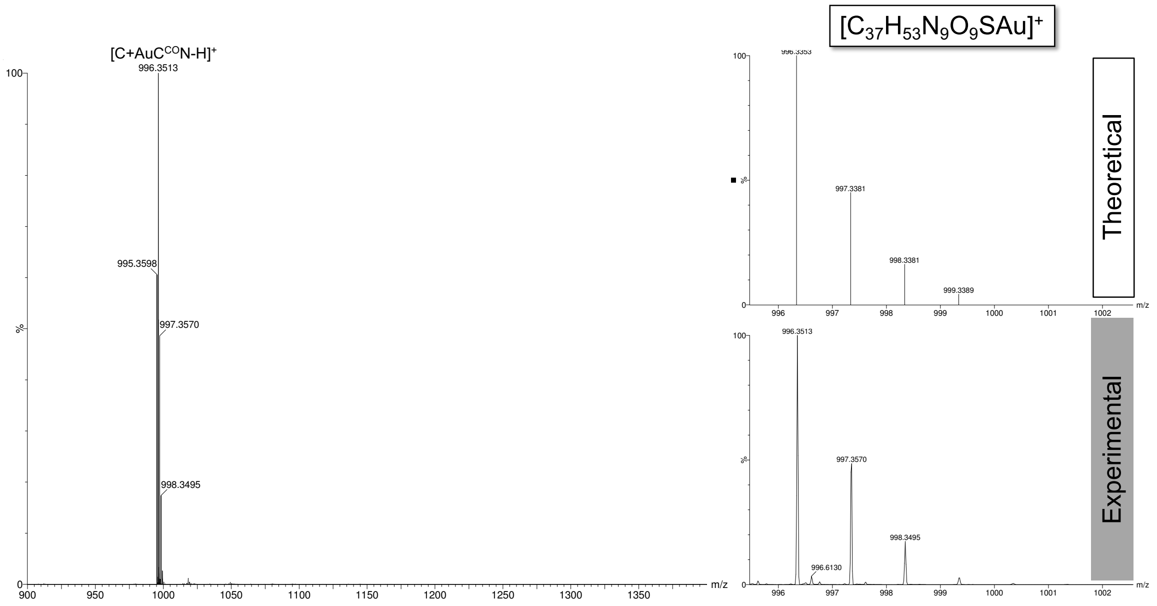

Figure S15 - HPLC-ESI-MS analysis of the reaction of compound **2** with C model peptide (1 : 1 ratio) after 30 min incubation at 37 °C recorded at retention time 5.29 min. Comparison between the experimental isotopic patterns of representative adducts with the theoretical values.

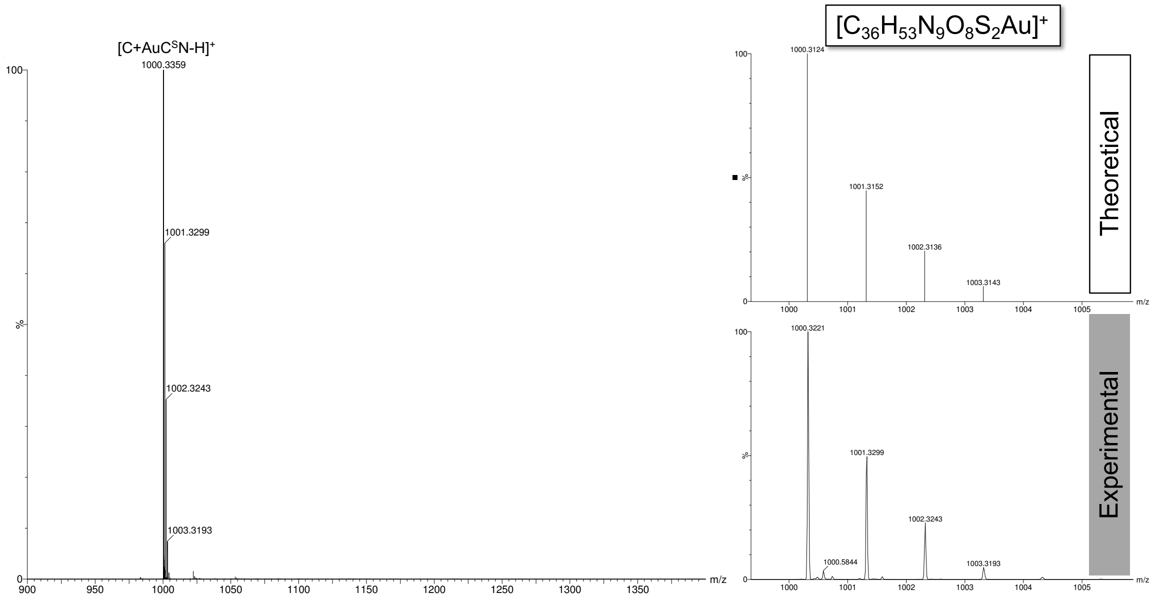

Figure S16 - HPLC-ESI-MS analysis of the reaction of compound **3** with C model peptide (1 : 1 ratio) after 30 min incubation at 37 °C recorded at different retention times (RTs). Comparison between the experimental isotopic patterns of representative adducts with the theoretical values.

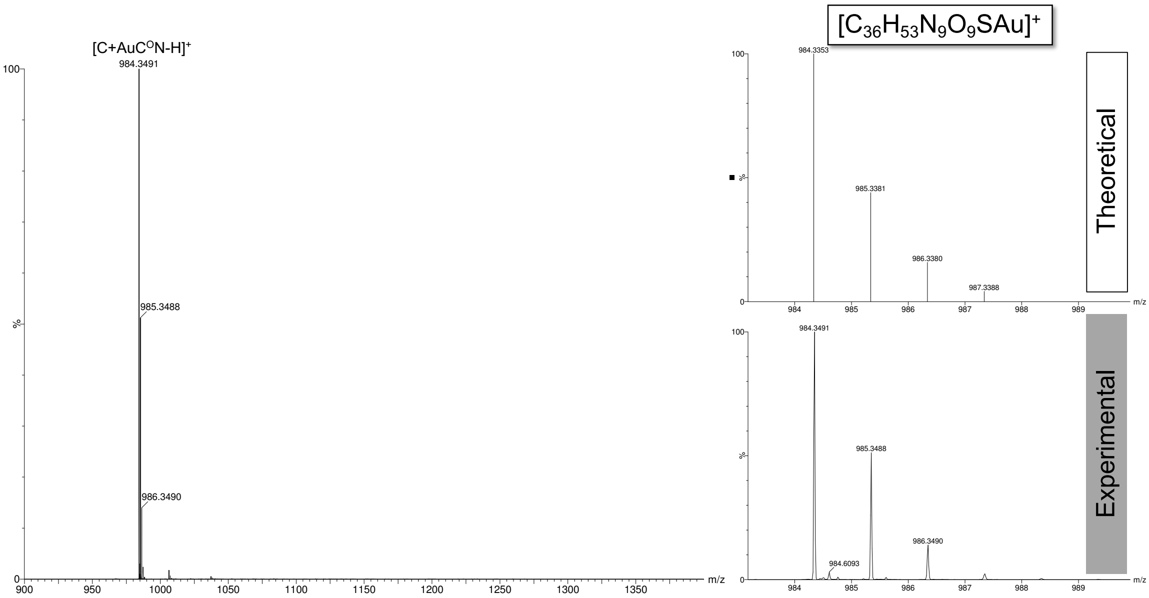

RT 5.19 min

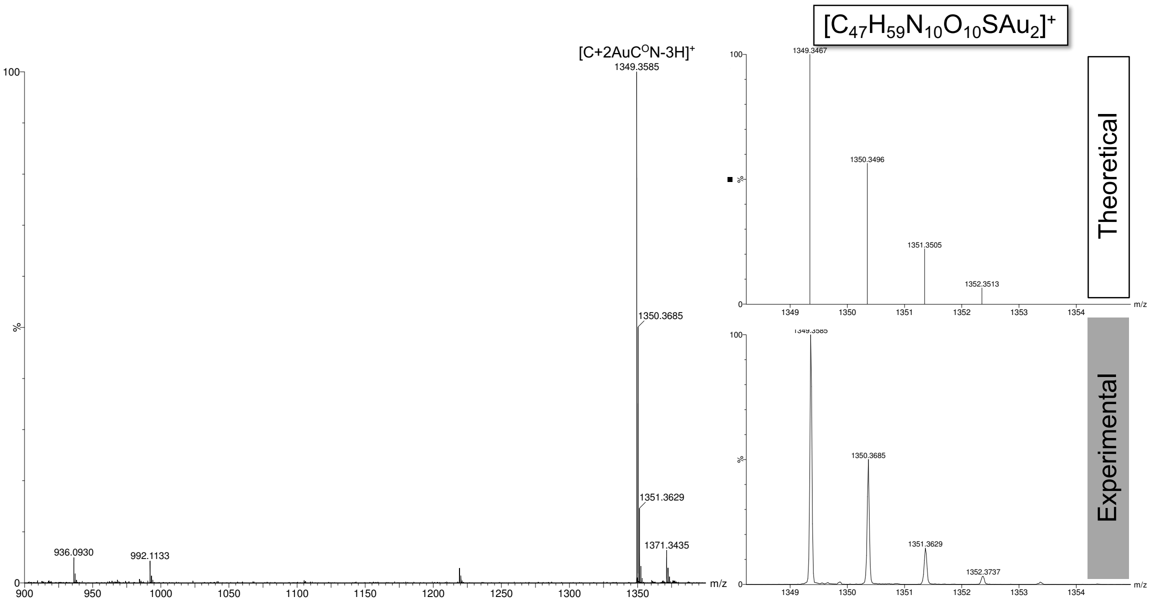

RT 6.90 min

Figure S17 - HPLC-ESI-MS analysis of the reaction of compound **4** with C model peptide (1 : 1 ratio) after 30 min incubation at 37 °C recorded at retention time 4.78 min. Comparison between the experimental isotopic patterns of representative adducts with the theoretical values.

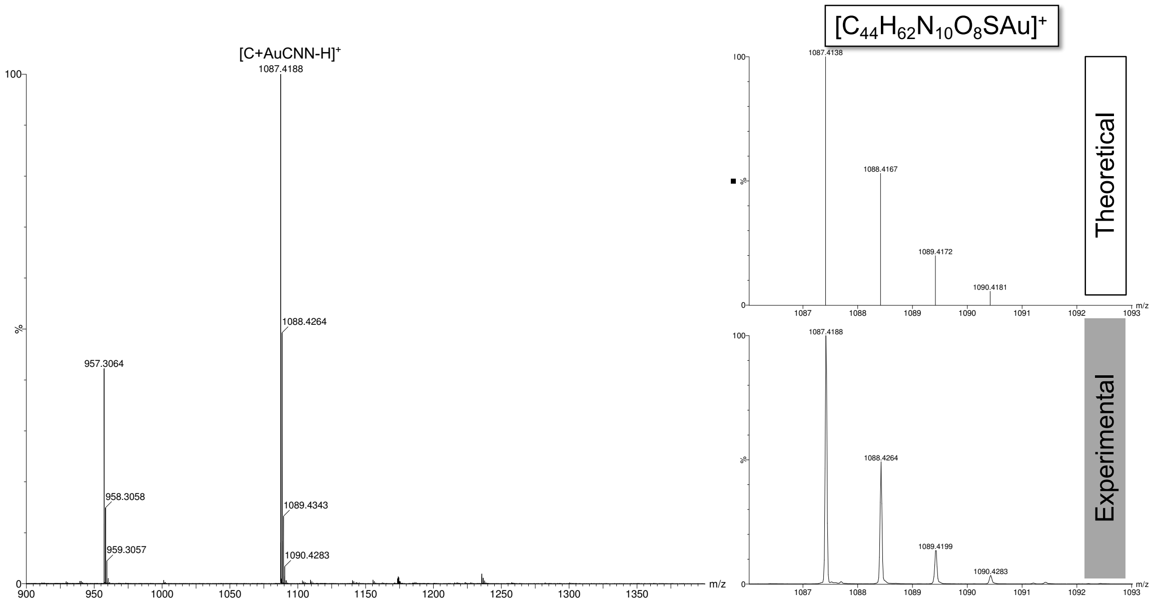

Figure S18 - HPLC-ESI-MS analysis of the reaction of compound **1** with GSH (3 : 1 ratio) after 24 h incubation at 37 °C. Comparison between the experimental isotopic patterns of representative adducts with the theoretical values.

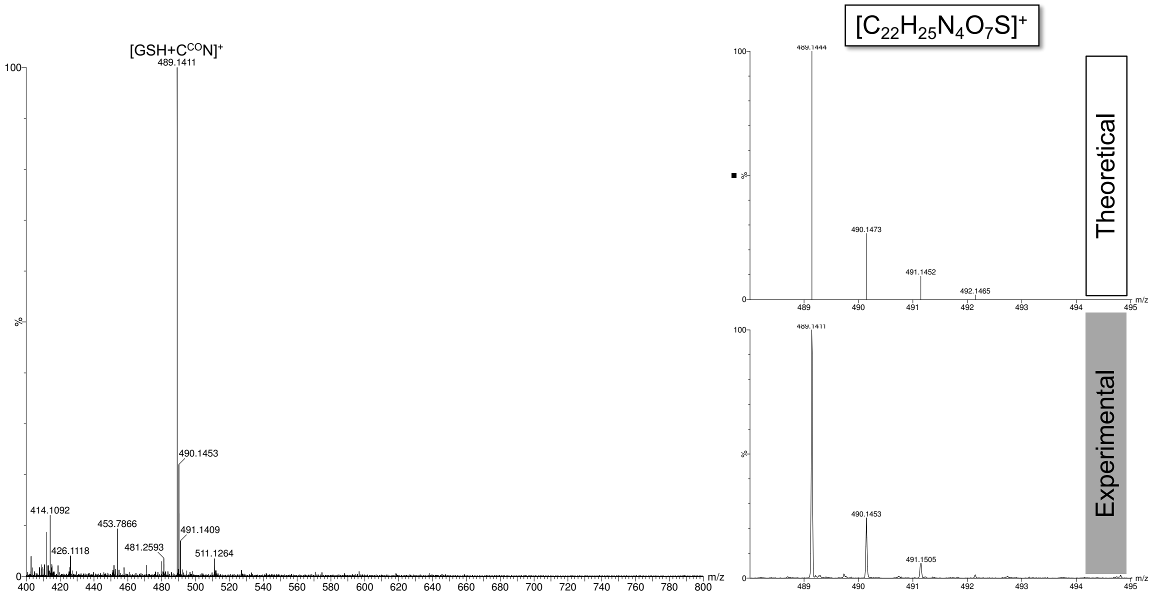

Figure S19 - HPLC-ESI-MS analysis of the reaction of compound **4** with GSH (3 : 1 ratio) after 24 h incubation at 37 °C. Comparison between the experimental isotopic patterns of representative adducts with the theoretical values.

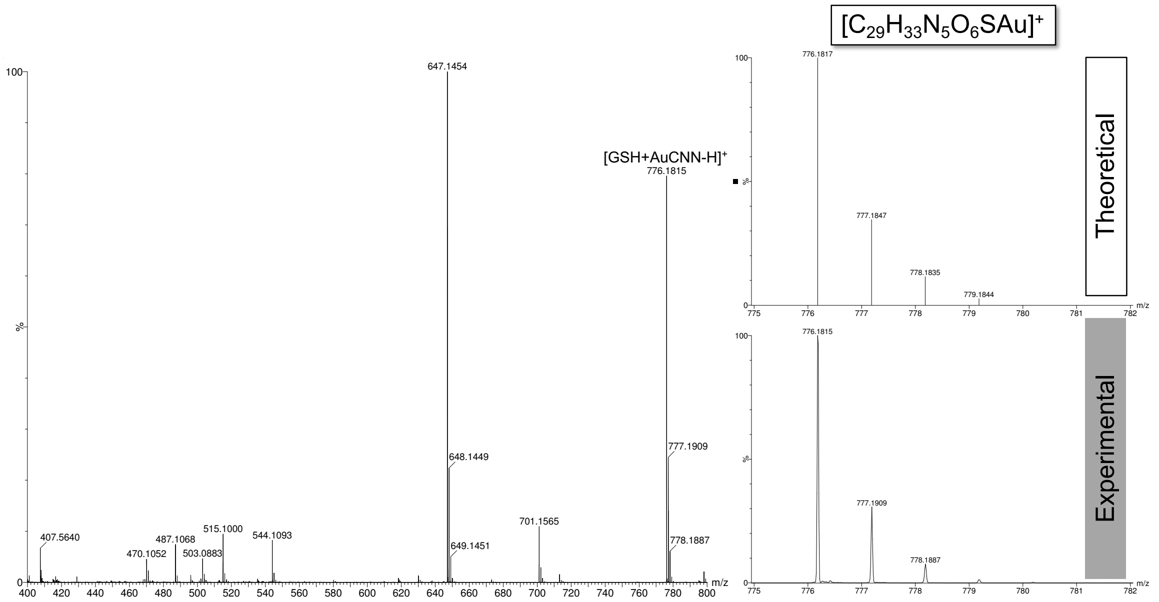

Figure S20 - HPLC-ESI-MS analysis of the reaction of compound **1** with L model peptide (3 : 1 ratio) after 30 min incubation at 37 °C recorded at retention time 4.37 min. Comparison between the experimental isotopic patterns of representative adducts with the theoretical values.

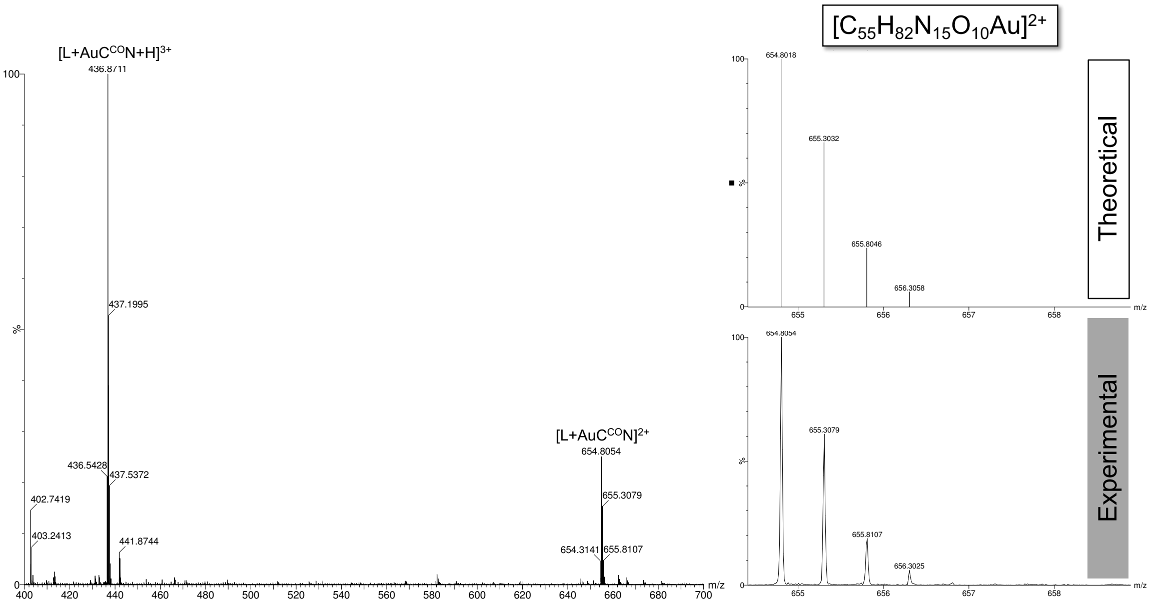

Figure S21 - HPLC-ESI-MS analysis of the reaction of compound **2** with L model peptide (3 : 1 ratio) after 30 min incubation at 37 °C recorded at retention time 4.51 min. Comparison between the experimental isotopic patterns of representative adducts with the theoretical values.

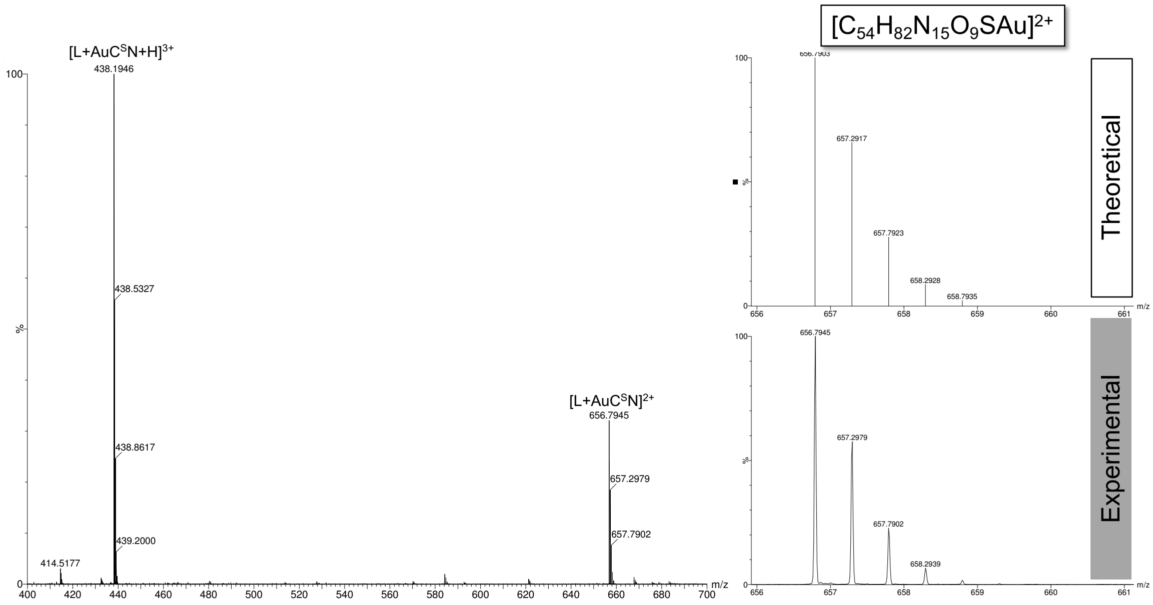

Figure S22 - HPLC-ESI-MS analysis of the reaction of compound **3** with L model peptide (3 : 1 ratio) after 30 min incubation at 37 °C recorded at retention time 4.39 min. Comparison between the experimental isotopic patterns of representative adducts with the theoretical values.

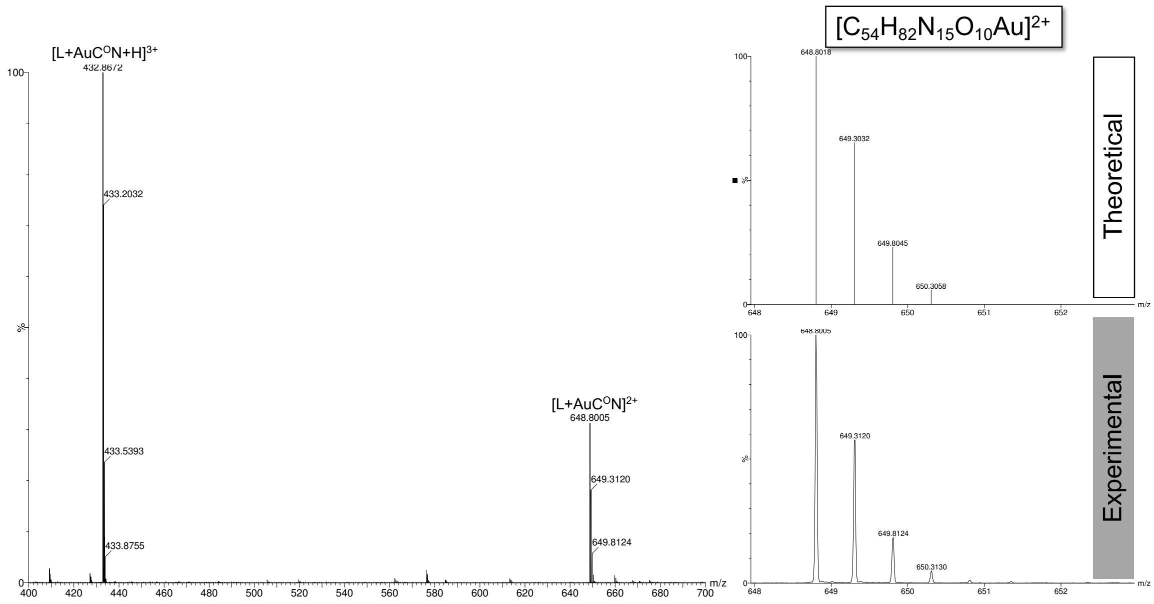

Figure S23 - HPLC-ESI-MS analysis of the reaction of compound **1** with LE model peptide (3 : 1 ratio) after 30 min incubation at 37 °C recorded at retention time 6.62 min. Comparison between the experimental isotopic patterns of representative adducts with the theoretical values.

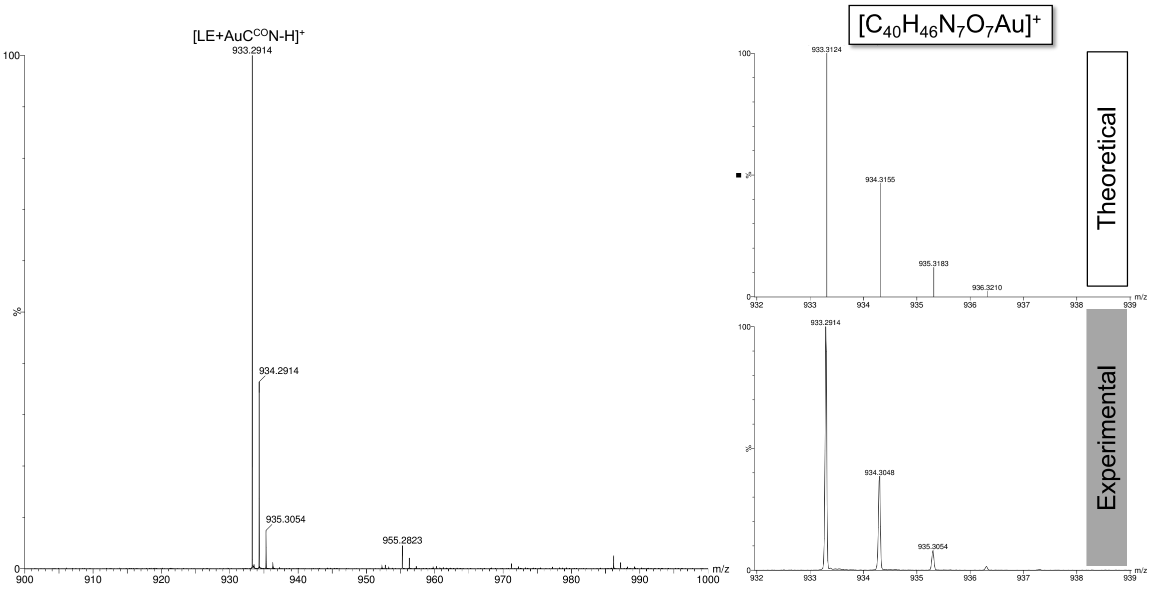

Figure S24 - HPLC-ESI-MS analysis of the reaction of compound **2** with LE model peptide (3 : 1 ratio) after 30 min incubation at 37 °C recorded at retention time 5.44 min. Comparison between the experimental isotopic patterns of representative adducts with the theoretical values.

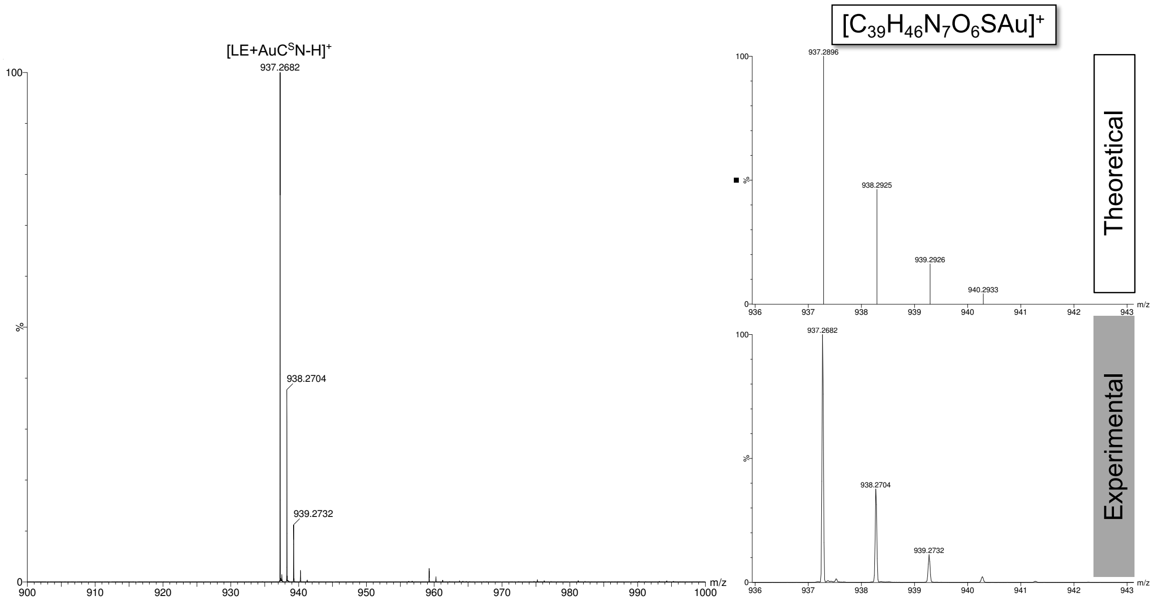

Figure S25 - HPLC-ESI-MS analysis of the reaction of compound **3** with LE model peptide (3 : 1 ratio) after 30 min incubation at 37 °C recorded at retention time 6.84 min. Comparison between the experimental isotopic patterns of representative adducts with the theoretical values.

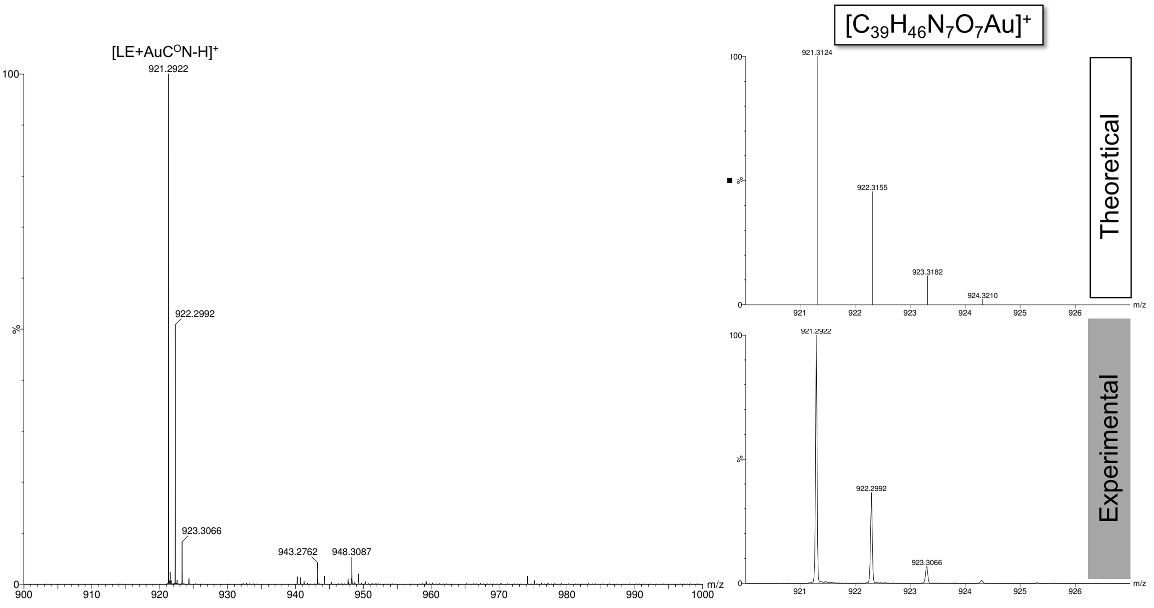

Figure S26 - Fragment-mass spectrum of the [LE+Au^{III}C^N-2H$^+$]$^+$ adduct (*m/z* 933) showing the identified metalated a-, b- (N-terminal) and ay- (internal) fragments. The fragmentation positions and the most likely binding sites (golden circles) are indicated in the structure of LE in the top right corner.

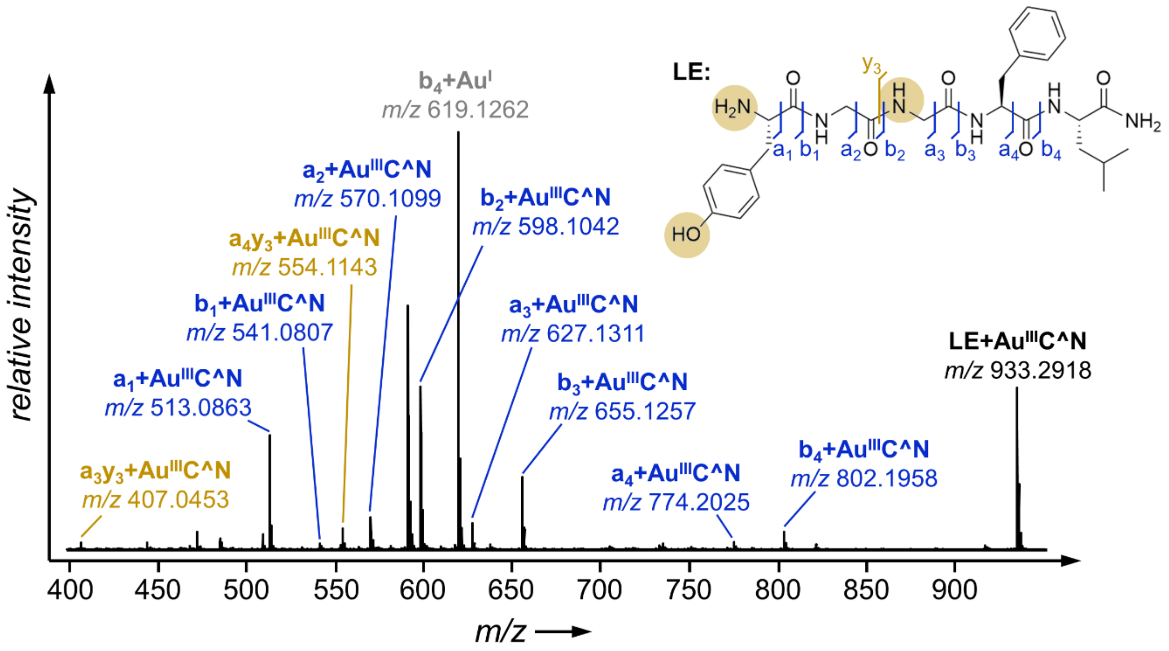

Figure S27 - HPLC-ESI-MS competition experiment: A) Chromatograms of the mixture of free peptides ZF and LE before (bottom) and after treatment (top) with compound **1**; B) mass spectrum of compound **1** reacting with both ZF Cys_2His_2 and LE in a 3:1:1 ratio for 30 min incubation at 37 °C; C) comparison between the experimental isotopic patterns of the $[Apo\text{-}ZF+Au^{III}C^{CO}N+3H]^{5+}$ adduct with the theoretical values.

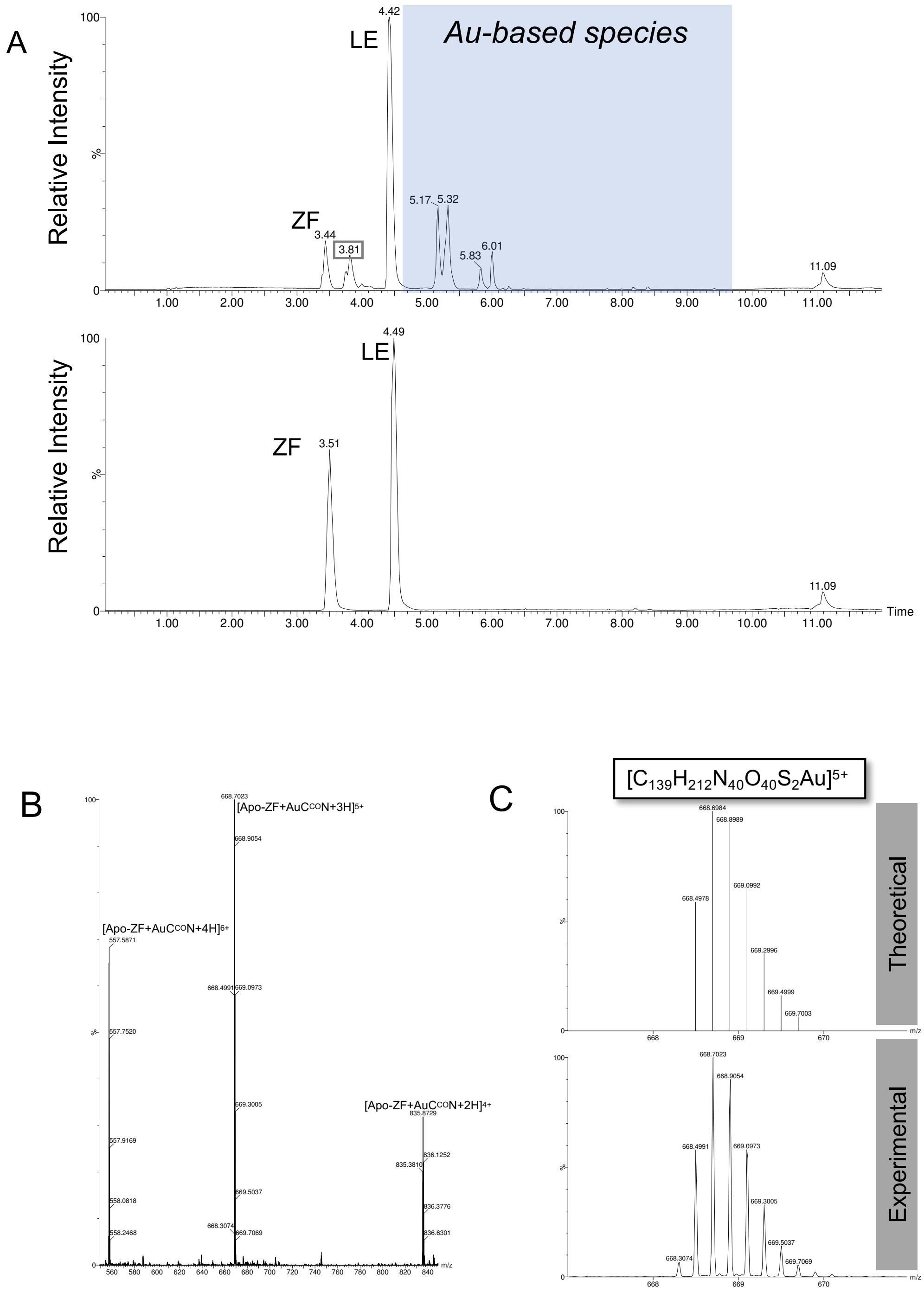

Figure S28 - Structures of the amino acid ligands obtained by DFT calculations and used to evaluate the standard formation Gibbs free energy of the corresponding Au(C^{CO}N)Cl adducts (Eq. 1).

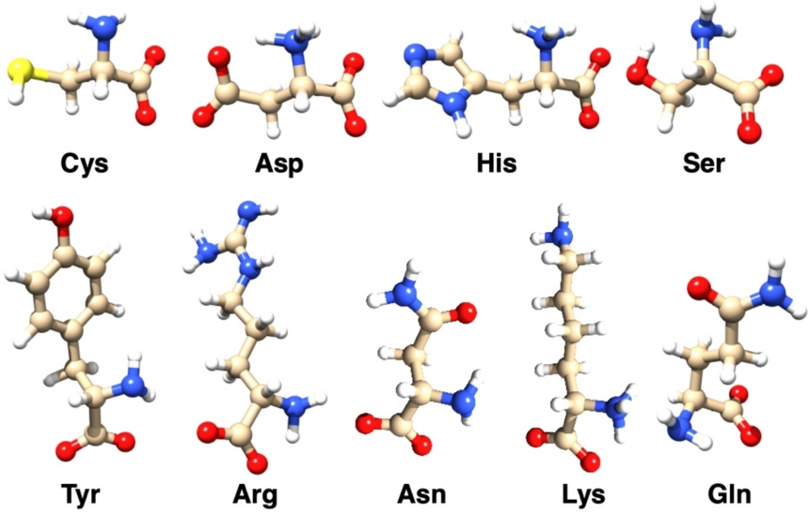

Figure S29 - Structures of the Au(C^{CO}N)Cl amino acid adducts obtained by DFT calculations. Their corresponding standard formation Gibbs free energy values (in kJ/mol, Eq. 1) are also indicated.

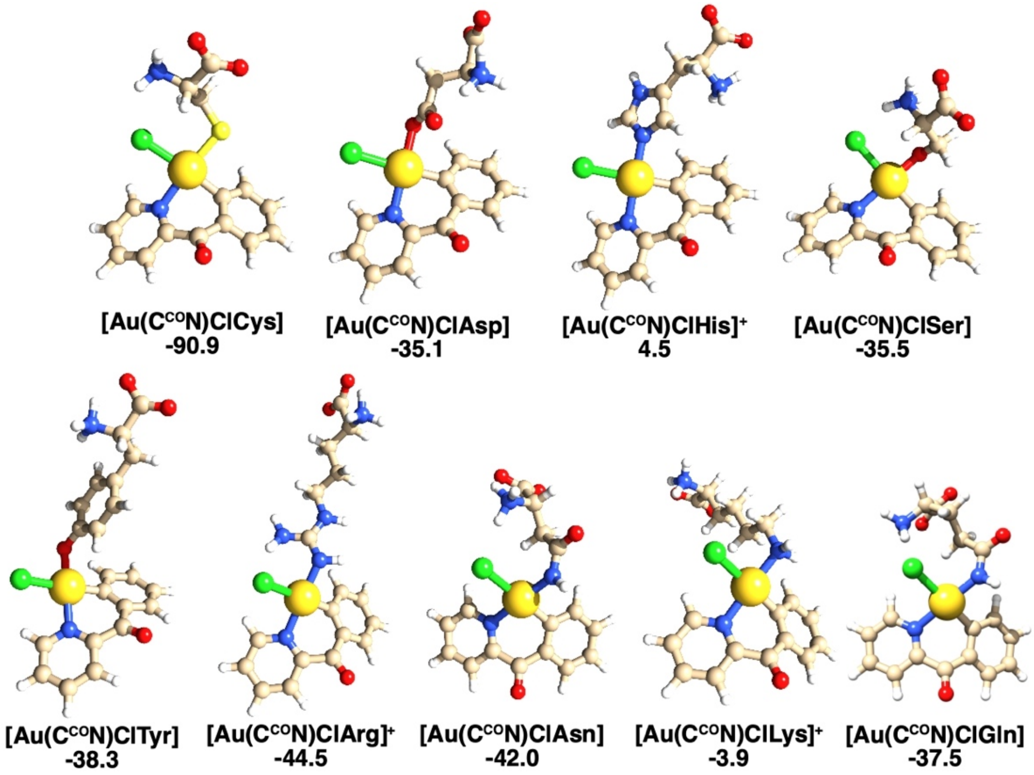

Figure S30 - Conformers and relative energy values (kJ/mol) of the cross-coupling reaction product of GSH with the (C^{CO}N) ligand, obtained by DFT calculations.

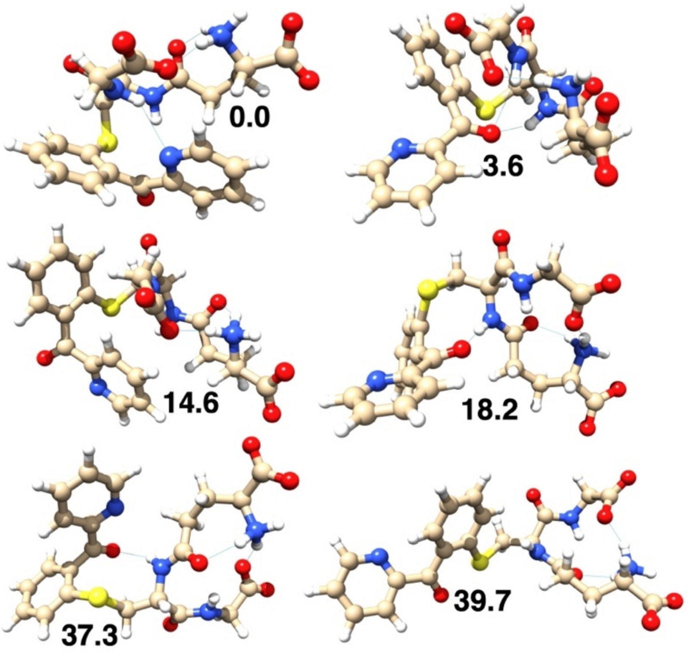

Figure S31 - Conformers and relative energy values (kJ/mol) of the cross-coupling reaction product of GSH with the ($C^{S}N$) ligand, obtained by DFT calculations.

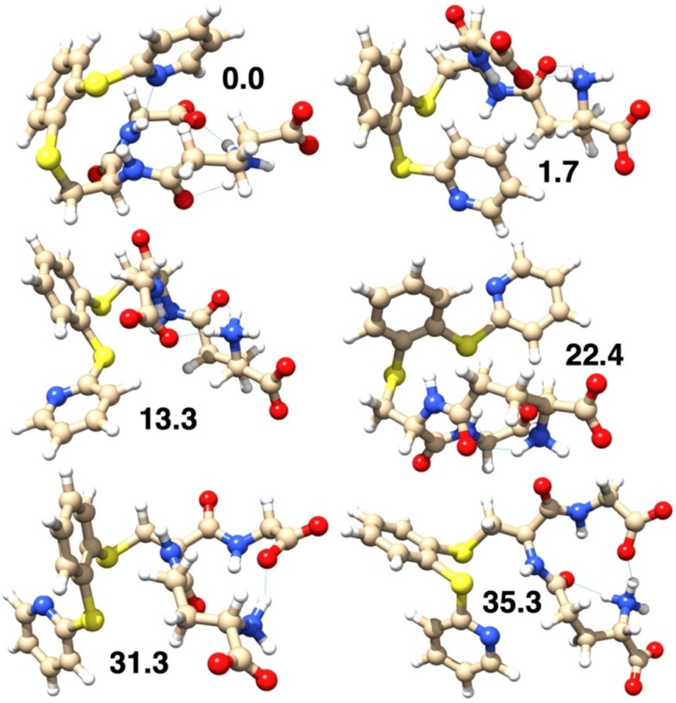

References

[1] aY. Fuchita, H. Ieda, A. Kayama, J. Kinoshita-Nagaoka, H. Kawano, S. Kameda, M. Mikuriya, *Journal of the Chemical Society, Dalton Transactions* **1998**, 4095-4100; bY. Fuchita, H. Ieda, Y. Tsunemune, J. Kinoshita-Nagaoka, H. Kawano, *Journal of the Chemical Society - Dalton Transactions* **1998**, *2*, 791-796;

cX. Ma, Q. Liu, X. Jia, C. Su, Q. Xu, *RSC Advances* **2016**, *6*, 56930-56935; dG. Marcon, S. Carotti, M. Coronnello, L. Messori, E. Mini, P. Orioli, T. Mazzei, M. A. Cinellu, G. Minghetti, *Journal of Medicinal Chemistry* **2002**, *45*, 1672-1677.

[2] aA. Laskay, C. Garino, Y. O. Tsybin, L. Salassa, A. Casini, *Chemical Communications* **2015**, *51*, 1612-1615; bM. N. Wenzel, R. Bonsignore, S. R. Thomas, D. Bourissou, G. Barone, A. Casini, *Chemistry - A European Journal* **2019**, *25*, 7628-7634.

[3] R. Bonsignore, S. R. Thomas, W. T. Klooster, S. J. Coles, R. L. Jenkins, D. Bourissou, G. Barone, A. Casini, *Chemistry - A European Journal* **2020**, *26*, 4226-4231.

[4] Y. Zhao, D. G. Truhlar, *J. Chem. Phys.* **2006**, *125*, 194101-194101.

[5] aL. E. Roy, P. J. Hay, R. L. Martin, *Journal of Chemical Theory and Computation* **2008**, *4*, 1029-1031; bK. L. Schuchardt, B. T. Didier, T. Elsethagen, L. Sun, V. Gurumoorthi, J. Chase, J. Li, T. L. Windus, *Journal of Chemical Information and Modeling* **2007**, *47*, 1045-1052.

[6] aR. Krishnan, J. S. Binkley, R. Seeger, J. A. Pople, *J. Chem. Phys.* **1980**, *72*, 650-654; bA. D. McLean, G. S. Chandler, *J. Chem. Phys.* **1980**, *72*, 5639-5648.

[7] J. Tomasi, B. Mennucci, R. Cammi, *Chemical Reviews* **2005**, *105*, 2999-3093.

[8] C. Peng, H. Bernhard Schlegel, *Israel Journal of Chemistry* **1993**, *33*, 449-454.

[9] A. K. Rappé, C. J. Casewit, K. S. Colwell, W. A. Goddard, W. M. Skiff, *Journal of the American Chemical Society* **1992**, *114*, 10024-10035.

[10] M. J. Frisch, G. W. Trucks, H. B. Schlegel, G. E. Scuseria, M. A. Robb, J. R. Cheeseman, G. Scalmani, V. Barone, B. Mennucci, G. A. Petersson, H. Nakatsuji, M. Caricato, X. Li, H. P. Hratchian, A. F. Izmaylov, J. Bloino, G. Zheng, J. L. Sonnenberg, M. Hada, M. Ehara, K. Toyota, R. Fukuda, J. Hasegawa, M. Ishida, T. Nakajima, Y. Honda, O. Kitao, H. Nakai, T. Vreven, J. A. Montgomery, J. E. Peralta, F. Ogliaro, M. Bearpark, J. J. Heyd, E. Brothers, K. N. Kudin, V. N. Staroverov, R. Kobayashi, J. Normand, K. Raghavachari, A. Rendell, J. C. Burant, S. S. Iyengar, J. Tomasi, M. Cossi, N. Rega, J. M. Millam, M. Klene, J. E. Knox, J. B. Cross, V. Bakken, C. Adamo, J. Jaramillo, R. Gomperts, R. E. Stratmann, O. Yazyev, A. J. Austin, R. Cammi, C. Pomelli, J. W. Ochterski, R. L. Martin, K. Morokuma, V. G. Zakrzewski, G. A. Voth, P. Salvador, J. J. Dannenberg, S. Dapprich, A. D. Daniels, Farkas, J. B. Foresman, J. V. Ortiz, J. Cioslowski, D. J. Fox, *Gaussian 09, Revision A.1, Gaussian, Inc., Wallingford CT* **2009**.

[11] E. F. Pettersen, T. D. Goddard, C. C. Huang, G. S. Couch, D. M. Greenblatt, E. C. Meng, T. E. Ferrin, *Journal of Computational Chemistry* **2004**, *25*, 1605-1612.
